# Environmental distribution, physiology and genomic adaptations of arctic ammonia-oxidizing archaea

**DOI:** 10.3389/fmicb.2026.1722591

**Published:** 2026-03-09

**Authors:** Marina Montserrat Díez, Maximilian Dreer, Christa Schleper, Melina Kerou

**Affiliations:** Department of Functional and Evolutionary Ecology, University of Vienna, Vienna, Austria

**Keywords:** ammonia-oxidizing archaea, arctic, climate change, nitrification, nitrous oxide, Nitrososphaeria, Thaumarchaeota

## Abstract

Ammonia oxidizing archaea (AOA) are the main drivers of nitrification in Arctic soils, ecosystems that are highly sensitive to climate change-induced warming and permafrost thaw, processes that may enhance nitrogen mobilization from the soil and increase emissions of the greenhouse gas nitrous oxide (N_2_O). Compared to other terrestrial environments, the diversity of AOA in arctic soils was reported to be very low, with only two specific clades detected in most arctic ecosystems. However, to date no ammonia oxidizing model organism was available in pure culture to study the effect of climate change on the arctic nitrifier communities. AOA diversity in Arctic soils has been considered low, typically dominated by two clades. However, the lack of a pure Arctic AOA isolate has constrained mechanistic understanding of how Arctic nitrifier communities respond to warming. In this study we assessed AOA diversity across soils spanning approximately half of the circumpolar Arctic by linking 16S rRNA and amoA gene taxonomies to improve clade-level resolution. We corroborated the widespread occurrence of the AOA clades NS-zeta and NS-gamma, while a third, non-ammonia oxidizing clade of *Nitrososphaerales* was also found to dominate specific sites suggesting putative roles in Arctic carbon cycling. These clades exhibited distinct distribution patterns and were differentially associated with soil physicochemical parameters such as pH, water content and organic carbon content. Furthermore, after 15 years of continuous cultivation, we isolated the first psychrotolerant Arctic AOA in pure culture and obtained its closed genome. The strain, *Candidatus* Nitrosocosmicus arcticus Kfb actively oxidized ammonia at 4 °C, the lowest temperature reported to date, extending the temperature range of reported ammonia oxidation from 4 °C to 74 °C. However, ammonia oxidation became unstable at temperatures above 20°C during prolonged incubation, indicating potential vulnerability to episodic warming. Together, these findings expand current understanding of Arctic AOA diversity and provide a genomic and physiological framework for investigating the response of Arctic nitrification processes to ongoing climate change.

## Introduction

Arctic soils act as global sinks of organic matter due to low temperatures, storing over 1,500 Pg of organic carbon (Koven et al., 2011), and 66 Pg of nitrogen (Harden et al., 2012). The ongoing rise in temperatures associated with climate change is exacerbating the risk of this reservoir becoming available to the decomposing activity of soil microorganisms, leading to the emission of the greenhouse gases carbon dioxide (CO_2_), methane (CH_4_) and N_2_O. This would generate a positive feed-back effect that would further aggravate the temperature increase (Hugelius et al., 2020; Koven et al., 2011; Voigt et al., 2017).

Nitrogen is a vital element that regulates key components of the carbon cycle at high latitudes (Chen et al., 2018; Mack et al., 2004); however, nitrogen availability for organisms is generally low in arctic soils (Nordin et al., 2004; Shaver and Chapin, 1980; Sistla et al., 2012). Paradoxically, arctic soils represent a significant source of N_2_O (Repo et al., 2009; Marushchak et al., 2011; Siljanen et al., 2019; Voigt et al., 2020), an ozone-depleting greenhouse gas 300 times more powerful than CO_2_ (Ravishankara et al., 2009; Intergovernmental Panel on Climate Change, 2014) produced through the activity of ammonia oxidizing microorganisms under aerobic and anaerobic conditions, or denitrifying microorganisms mostly under anaerobic conditions (Morley et al., 2008; Baggs, 2011). Consequently, the study of the N cycle is essential to understand the nutrient fluxes and predicting shifting ecosystem dynamics in a permafrost climate feedback scenario (Strauss et al., 2022).

Ammonia oxidizers, comprising ammonia oxidizing bacteria (AOB), comammox and ammonia oxidizing archaea (AOA), perform the first step of nitrification with the oxidation of ammonia to nitrite. The ammonia oxidation process in comammox is a one-step process that directly converts ammonia into nitrate. The resulting nitrite (NO₂^−^) is not expelled from the cell, but is immediately captured by intracellular nitrite oxidoreductase (Nxr) and oxidized into the final product - nitrate (NO₃^−^). In arctic soils AOA have been shown to be the most abundant ammonia oxidizers with demonstrated activity, while AOB are less prevalent or even undetectable (Alves et al., 2013; Siljanen et al., 2019). Thus, N_2_O emissions are mostly associated with AOA activity, either directly by production through abiotic reactions involving intermediate compounds from the ammonia oxidation process (Kozlowski et al., 2016), in some clades enzymatically (Jung et al., 2019), or indirectly by supplying the necessary substrates for other denitrifying microorganisms (Siljanen et al., 2019).

AOA are a ubiquitous and functionally heterogeneous group, comprising multiple lineages or clades within the order *Nitrososphaerales* that exhibit distinct ecophysiologies (Abby et al., 2020; Alves et al., 2018; Herbold et al., 2017; Kerou et al., 2021; Jung et al., 2022; Dreer et al., 2025). The main terrestrial AOA clades include the NS-alpha, NS-zeta, NS-gamma, NS-delta, NS-beta and NS-epsilon clades within the *Nitrososphaeraceae*, and NT-alpha and NP-eta clades within the *Nitropumilaceae*. In particular, the NS-zeta clade (*Candidatus* Nitrosocosmicus sp.) is distinguished by a set of features including low substrate affinity for ammonia (Jung et al., 2022), growth with aggregate formation (Jung et al., 2016; Liu et al., 2019), robust biofilm-forming capacity (Dreer et al., 2025) and the absence of an S-layer (Han et al., 2024). Moreover, NS-zeta is among the few AOA terrestrial clades with cultivated representatives, together with NS-alpha and NT-alpha (Tourna et al., 2011; Lehtovirta-Morley et al., 2014; Jung et al., 2016). canonical terrestrial AOA lineages, several related clades within the Nitrososphaerales occur in soils but lack the genetic capacity for ammonia oxidation. These microorganisms - often termed non-ammonia-oxidizing archaea (non-AOA) - are represented by distinct phylogenetic clades, including the mesophilic group 1.1c, currently classified within the *Ca.* Gagatemarchaeaceae family (Sheridan et al., 2023), and group 1.3 (Biggs-Weber et al., 2020). Although the only cultivated representative reported to date was isolated from an acidic hot spring and shown to reduce iron and sulfur capacity (Kato et al., 2021), non-AOA *Nitrososphaerales* can nevertheless constitute a prevalent archaeal group in top soil and sub soil horizons (Kemnitz et al., 2007; Lehtovirta et al., 2009; Oton et al., 2016).

In arctic soils, phylogenetic diversity of AOA is generally low, mostly confined to the terrestrial clades NS-zeta and NS-gamma (Alves et al., 2013; Siljanen et al., 2019). Our understanding of the physiological capabilities that enable only selected AOA to thrive in such unique environments is limited by the fact that the only reported AOA culture from arctic soils is the enrichment culture of *Ca.* Nitrosocosmicus arcticus Kfb, belonging to the NS-zeta clade, with an incomplete genome (Alves et al., 2019). This strain is characterized by its slow growth rate compared to the other mesophilic cultivated AOA, lower optimal growth temperature (stable growth at 20 °C), and strong tendency toward aggregate formation in liquid culture. On the other hand, NS-gamma, although being one of the most frequently detected AOA clades in the environment (Alves et al., 2018), remains uncultivated and is so far represented by a handful of metagenome-assembled genomes (MAGs) from polar regions which comprise the genus *Ca.* Nitrosopolaris (Pessi et al., 2022).

Due to inherent difficulties in accessing arctic ecosystems, there are many open questions regarding the main nitrifier populations in the arctic as well as the physiological capabilities that enable AOA to thrive in such unique environments. In this study, we investigated the environmental distribution of AOA clades across the arctic circumpolar region in order to expand previous analyses of selected localities. Additionally, we report the isolation in pure culture and the resequencing and genome closure of *Ca.* Nitrosocosmicus arcticus strain Kfb, the only available psychrotolerant AOA isolate from arctic soils, extending the original characterization of its physiology and genome properties (Alves et al., 2019). We aimed to establish associations between the observed environmental patterns of distribution and specific functions identified by comparative genomics of both clades in order to uncover common ecophysiological traits in the arctic AOA clades.

## Materials and methods

### Description of the study sites and sample preparation

The soil samples used in the present study were collected between 2007 and 2012, in the frame of the CryoCARB project, by the project research associates from 4 different locations: Cherskii (Republic of Sakha, Russia) (Gittel et al., 2014), Zackenberg (Greenland) (Gittel et al., 2014), Taymyr Peninsula (Central Siberia), and Tazovsky (Western Siberia) (Gentsch et al., 2015). These four locations are found along the arctic circumpolar area and the dominant vegetation type is tundra. At each location, several sites were selected based on their dominant vegetation subtypes and the samples were collected from all major soil horizons within each profile. In this study, we pooled together the organic topsoil samples (i.e., O horizon) belonging to the same location site and vegetation subtype (summarized in Supplementary Table S1). In addition, we also included in our study the samples belonging to the buried topsoil fractions (i.e., Ajj horizon), resulting in 15 samples in total. Sites, vegetation types and nucleic acid extractions were described previously (Gittel et al., 2014; Gittel et al., 2014; Gentsch et al., 2015), and original extractions and dilutions were stored at −80 °C and −20 °C since then. Soil data available for the different locations were compiled from the original sources and from additional studies (Wild et al., 2013; Gentsch et al., 2018) (Supplementary Table S1). For this study, we calculated the averages of the selected environmental parameters, using the individual values of the samples that were used to make each pool of samples (Supplementary Tables S2, S3).

### Nested PCR amplification and high throughput amplicon sequencing

As our initial approach of generating *amoA* gene amplicons was unsuccessful, we opted for a nested PCR approach on the 16S rRNA gene. By amplifying the archaeal signal in 16S rRNA gene amplicons (Pausan et al., 2019), we aimed to enrich the AOA fraction, which were expected to be in very low abundance in arctic soils. The primer pair combinations used were the following: first PCR 344f (5’-ACGGGGYGCAGCAGGCGCGA-3′) - 1041R (5′-GGCCATGCACCWCCTCTC-3′), and second PCR Arch-519F (5’-CAGCCGCCGCGGTAA-3′) - 806R (5’-GGACTACHVGGGTWTCTAAT-3′). For the first PCR round, reactions were carried out in triplicates of 50 ul reaction volume containing: 0.4 U Phire Hot Start II DNA Polymerase (ThermoFisher), 1x Phire Reaction Buffer, 0.2 mM dNTPs, 3% DMSO, 0.5 μM of each primer. The cycling conditions were the following: 3 min initial denaturation step at 98 °C, followed by 25 cycles of 10 s denaturing at 98 °C, 30 s annealing at 56 °C and 30 s extension at 72 °C, with a final extension step of 5 min at 72 °C. Pooled triplicate PCR products were column-purified with the Monarch® PCR & DNA Cleanup Kit (New England BioLabs, US). The second PCR round and the sequencing were performed at the sequencing facility Novogene (UK), using the Illumina NovaSeq Paired-End platform (2 × 250 bp).

### Sequence processing and *amoA* clade assignment using a 16S-*amoA* database

Adaptor and primer sequences from the 16S rRNA gene reads were trimmed with CUTADAPT (Martin, 2011). Subsequently, the amplicons were processed with the DADA2 pipeline (Callahan et al., 2016) in R v. 4.2.1. The forward and reverse reads were truncated at positions 200 and 200, respectively, and the low quality reads and chimeras were filtered out. The resulting Amplicon Sequencing Variants (ASVs) were taxonomically assigned by the RDP classifier using the GTDB v. 202 database (Ali, 2024). In order to obtain the specific *amoA* gene based taxonomy for AOA (Alves et al., 2018) from the 16S rRNA gene ASVs, we used a correspondence database (Wang et al., 2021). In the original study, 16S rRNA genes retrieved from publicly available AOA genomes, metagenomes and contigs on which the correspondent *amoA* gene was also encoded were assembled and a correspondence 16S-to-*amoA* clade database was built. The database (16S-*amoA* database) was updated with additional sequences for this study, resulting in 258 16S rRNA gene sequences linked to their corresponding 258 *amoA* genes (Supplementary material). We selected the ASVs classified as *Nitrososphaerales* and ran them against the 16S-*amoA* database using BLASTN, with a > 97% sequence similarity setting. The allocation of an ASV into an *amoA* clade occurred when at least two out of the three best hits matched a specific assignment.

### Phylogenetic analysis

*Nitrososphaerales* ASVs aligned to 220 AOA 16S rRNA full gene reference sequences representing the different *amoA* clades with the online tool MAFFT v. 7.490 (Katoh and Toh, 2008) (scoring matrix 1PAM/k = 2). The phylogenetic reconstruction was calculated by maximum-likelihood in IQTREE v. 2.2.0 (Trifinopoulos et al., 2016), with the TIM3e + I + G4 substitution model and 1,000 ultrafast bootstrap replicates (Minh et al., 2013). The tree visualization and annotation was performed with the online tool iTOL (Letunic and Bork, 2021).

Following the same procedure as before, we selected only the non-AOA *Nitrososphaerales*, aligned them to the reference 16S rRNA tree (Oton et al., 2016) and reconstructed the phylogeny (SYM + I + G4 substitution model). This was done in order to obtain the specific phylogenetic clades proposed for non-AOA in Oton et al. (2016) from the non-AOA ASVs.

### Statistical analysis

Downstream analysis and visualization were performed with the packages ampvis (Albertsen et al., 2015), phyloseq (McMurdie and Holmes, 2013) and vegan (Oksanen et al., 2025), rstatix (Kassambra, 2023) in R v. 4.2.1. To explore the potential relationships between the environmental parameters and the *amoA* clades in the arctic tundra soils, Canonical Correspondence Analysis (CCA) models were built with the vegan package. Using the Nitrososphaerales ASV count table, two models were constructed with soil physicochemical parameters and enzymatic activity values as environmental predictor matrices. The environmental parameters had been log transformed prior to the analysis, with the exception of the pH values. The CCA models were then tested for significance with a Monte Carlo permutation test, with the function anova.cca (vegan).

### Isolation and cultivation of *ca.* Nitrosocosmicus arcticus Kfb

*Ca.* N. arcticus Kfb (the strain indicator Kfb will be omitted from the rest of the manuscript for reading clarity) was originally isolated from arctic mineral gleysol frost boil soil situated in a tundra fen peatland in Knudsenheia, Svalbard, Norway (Alves et al., 2013). It was routinely grown in 30 mL polystyrene containers with a total volume of 20 mL at 20 °C in the dark without shaking. Growth medium consisted of fresh water medium (FWM: 17.11 mM NaCl, 1.97 mM MgCl2 6H2O, 680.23 μM CaCl2 2H2O, 6.71 mM KCl, 1.47 mM KH2PO4), supplemented with 1 mM NH4Cl, 2 mM NaHCO3, 1 mM pyruvate, 7.5 μM Fe-Na-EDTA, 0.1% v/v non-chelated trace element solution (Reyes et al., 2020). The starting pH of the growth medium was 7.3. Due to the stoichiometric conversion of ammonia to nitrite which coincides with cell growth (Könneke et al., 2005; Tourna et al., 2011; Lehtovirta-Morley et al., 2016), growth was followed by measuring nitrite concentrations colorimetrically as described before (Reyes et al., 2020). The standard inoculation volume was 20% v/v at nitrite concentrations between 600–700 μM. The remaining bacterial contaminants were eliminated by addition of a mix of 50 μg/mL of azithromycin (63.69 μM) and ciprofloxacin (150.90 μM). These antibiotics are known to be effective against biofilms and were successfully used to highly enrich *Ca.* Nitrosocosmicus sp. attached to quartz sand particles (Liu et al., 2019). Antibiotic stocks were prepared in water regardless of their known limited solubilities in water as AOA are sensitive to organic solvents (Stieglmeier et al., 2014; Sauder et al., 2016). The resulting antibiotic suspensions were mixed before addition to the growth medium. After two and four transfers in the selective medium, no bacterial contaminants were seen microscopically and no signal found when amplifying bacterial and fungal rRNA genes by PCR, respectively. The purity of cultures was routinely assessed by plating aliquots on R2A agar plates. The temperature range of *Ca.* N. arcticus was tested in the same medium and settings as described above. All temperature test cultures were inoculated from the same inoculum grown at 20 °C. To test the pH range of *Ca.* N. arcticus, 22 mL of buffered medium (growth medium supplemented with 10 mM buffer) were prepared and the starting pH measured with a pH meter. The following buffers were used to investigate the pH range of *Ca.* N. arcticus: MOPS (pH 6.5). HEPES (pH 7, 7.5, 8). Due to the low final buffer concentration used (10 mM), 1 M buffer stock solutions had to be titrated to pH values different from the desired final pH. To achieve final pH values of 6.5, 7.0, 7.5, and 8.0, stock solutions were titrated to 6.35, 7.06, 7.90, and 8.49, respectively.

### Imaging of *Ca.* Nitrosocosmicus arcticus

Cells were routinely imaged using phase contrast microscopy with 60x or 100x oil immersion objectives. Autofluorescence of *Ca.* N. arcticus was imaged using a mercury lamp and Nikon DAPI filter cube with exposure times between 50 and 200 ms.

To disturb aggregates of *Ca.* N. arcticus as little as possible, cells were prepared as follows for scanning electron microscopy using 1 mL wide bore tips for pipetting. Aggregates sedimenting at the bottom of growth containers were fixed in-situ for 5 min by adding glutaraldehyde to growth medium to a final concentration of 2.5%, before being transferred to PBS buffer containing 2.5% glutaraldehyde. Cells were washed in PBS 3x times and deposited onto a poly-l-lysine coated coverglass (0.1%) for 1 h. Cells were dried by an ethanol series (30, 50, 70, 80, 90, 100%) and subsequent critical point drying with CO_2_. Dry cells were sputter coated with Au and imaged with a JEOL IT 3000 at 20 kV.

### Nitrous oxide measurements of *Ca.* Nitrosocosmicus arcticus

The production of nitrous oxide was measured by growing 60 mL of *Ca.* N. arcticus in growth medium in 120 mL glass serum bottles sealed with blue butyl rubber stoppers. At ~500 μM nitrite 15 mL of the gas phase was transferred to sealed glass vials with a syringe and stored at room temperature in the dark until analysis. Nitrous oxide concentrations were determined with a Trace Gas Chromatograph (TRACE Ultra Gas Chromatograph, Thermo Scientific) equipped with a pulsed discharge ionization detector.

### High molecular weight (HMW) DNA extraction, sequencing and genome assembly of *Ca.* Nitrosocosmicus arcticus

HMW DNA of *Ca.* N. arcticus was isolated by a combination of gentle mechanical lysis followed by a phenol chloroform extraction and further enrichment of HMW DNA using magnetic beads.

A total of 1.5 liters of N. arcticus culture were concentrated by vacuum filtration [Nalgene reusable bottle top filters (Thermo Scientific)] using 0.22 μm mixed cellulose ester membrane filters (MF-Millipore), which were stored at −20 °C. Cells were washed of filters, resuspended in 0.5 mL modified TENST buffer (20 mM TrisHCl pH 8, 1 mM EDTA, 100 mM NaCl, 5% vol/vol sodium lauroylsarcosine, 0.12% Triton X_100) (Meulenbroek et al., 2013), transferred to safe screw tubes containing lysis matrix E (MP Biomedicals) and lysed in a bead beater (MP Biomedicals) for 30 s at 5 m/s. DNA was isolated from the lysate by a phenol-chloroform extraction (1x equal volume phenol:chloroform:isoamylalcohol 25:24:1, 1x equal volume chloroform:isoamylalcohol 24:1), centrifuging with 4,500 g for 3 min at 4 °C between steps, using only wide bore tips for pipetting and never vortexing samples. DNA was precipitated in 2x volumes of polyethylene glycol (PEG) 6,000 solution (30% weight/volume PEG6000, 1.6 M NaCl) containing 1 μL glycogen at 4 °C overnight. DNA was washed 3x in 4 °C 70% EtOH, centrifuging at 12,000 g for 30 min at 4 °C between steps, always leaving ~20 μL liquid when taking off supernatant as PEG DNA pellets are highly unstable. DNA was dried for a maximum of 15 min and resuspended in prewarmed 100 μL (60 °C) TE buffer (10 mM TrisHCl, 1 mM EDTA, pH 8) by finger flicking the tube as vortexing and or pipetting can shear HMW DNA. HMW DNA was further resuspended by incubation at 40 °C for 2 h and stored at either −20 °C or 4 °C for long or short term storage, respectively. RNA was digested with RNase A (1:10 vol/vol) for 1 h at 37 °C and samples checked for purity using Qubit Fluorometric Quantification assays (Thermo Scientific). Samples were enriched for HMW DNA using AMPure XP magnetic beads (Beckman Coulter) with a 0.6x ratio of beads to sample. Briefly, DNA is mixed with magnetic beads, the solution homogenized by finger flicking and DNA washed twice with 70% EtOH by adding the tubes to a magnetic rack. Finally, the HMW DNA is eluted in TE buffer or DEPC and transferred to DNA LoBind tubes. HMW DNA sequencing with a MinION nanopore sequencer and demultiplexing was performed by the Next Generation Sequencing Facility at Vienna BioCenter Core Facilities (VBCF), Vienna BioCenter (VBC), Austria.

Assembly was performed with flye v.2.9.5 in –nano_raw and –meta mode (Kolmogorov et al., 2019), obtaining a circularized genome. Basic genome statistics on the closed genome were performed with genometools v.1.6.2 (Gremme et al., 2013), followed by a taxonomic classification with gtdbtk v.2.4.0 (Chaumeil et al., 2022). To obtain a complete genome, we polished the draft genome first with medaka v. 2.1.1 (medaka: Sequence correction provided by ONT Research. https://github.com/rrwick/Unicycler. Accessed August 2025) followed by three rounds of Pilon v. 1.24 (Walker et al., 2014), using *Ca.* N. arcticus short reads generated in (Alves et al., 2019).

### Genome annotation and comparative genomics

Gene prediction and annotation was performed automatically using the Microscope annotation platform from Genoscope (Vallenet et al., 2019), followed by manual curation. Annotations were supplemented with arCOG assignments from the archaeal Clusters of Orthologous Genes database (2018 release, Galperin et al., 2025), Carbohydrate-active enzymes assignments (CAZymes) from dbCAN2 (v7.0) (Drula et al., 2022) and TCDB family assignments from the Transporter Classification Database (Saier et al., 2021).

Orthologous protein families from a dataset including the new assembly of *Ca.* N. arcticus, the closed genomes of *Ca.* N. franklandianus C13 (Lehtovirta-Morley et al., 2016), *Ca.* N. oleophilus MY3 (Jung et al., 2016), *Ca.* N. hydrocola G61 (Sauder et al., 2017), *Nitrososphaera viennensis* EN76 (Stieglmeier et al., 2014), *Ca.* Nitrososphaera evergladensis SR1 (Zhalnina et al., 2014), *Nitrosotalea devaniterrae* Nd1 (Lehtovirta-Morley et al., 2024), and the candidatus taxa based on MAGs Nitrososphaeraceae archaeon RRmetagenome_bin19 (*Ca.* Nitrosopolaris wilkensis) (Ji et al., 2017), Nitrososphaeraceae archaeon UBA347 (*Ca.* Nitrosopolaris nunavutensis UBA347) (Parks et al., 2017), Nitrososphaeraceae archaeon UBA536 (N. nunavutensis UBA536) (Parks et al., 2017) and Nitrososphaeraceae archaeon S100_Bin_4 (*Ca.* N. rasttigaisensis) (Pessi et al., 2022) were constructed using Orthofinder (v.2.5.4) (Emms and Kelly, 2019) with standard settings.

## Results

### Environmental distribution of *Nitrososphaerales* in arctic circumpolar tundra soils

In order to investigate AOA diversity, we sequenced the 16S rRNA gene using a nested primer pair combination targeting archaea (“Materials and Methods”) on 15 samples from four different locations across the Arctic circumpolar area (Supplementary Figure S1; Supplementary Table S1). Processing of the raw reads with DADA2 resulted in 950,188 amplicon reads, with a median of 62,403 reads per sample, from which 4,563 ASVs were recovered in total. From these, 803 ASVs were taxonomically assigned to Archaea and 3,744 ASVs to Bacteria, while in terms of relative abundance, 78.52% counts corresponded to Archaea and 18.37% to Bacteria. The most frequently detected archaeal phyla were *Thermoproteota* (2.4–78%), *Nanoarchaeota* (3.7–49.5%), *Thermoplasmatota* (0.01–38%) and *Methanobacteriota* (0.13–56.5%) (Supplementary Figure S2). Virtually all the *Thermoproteota* reads (99.5%) were classified within the order *Nitrososphaerales*.

**Figure 1:**
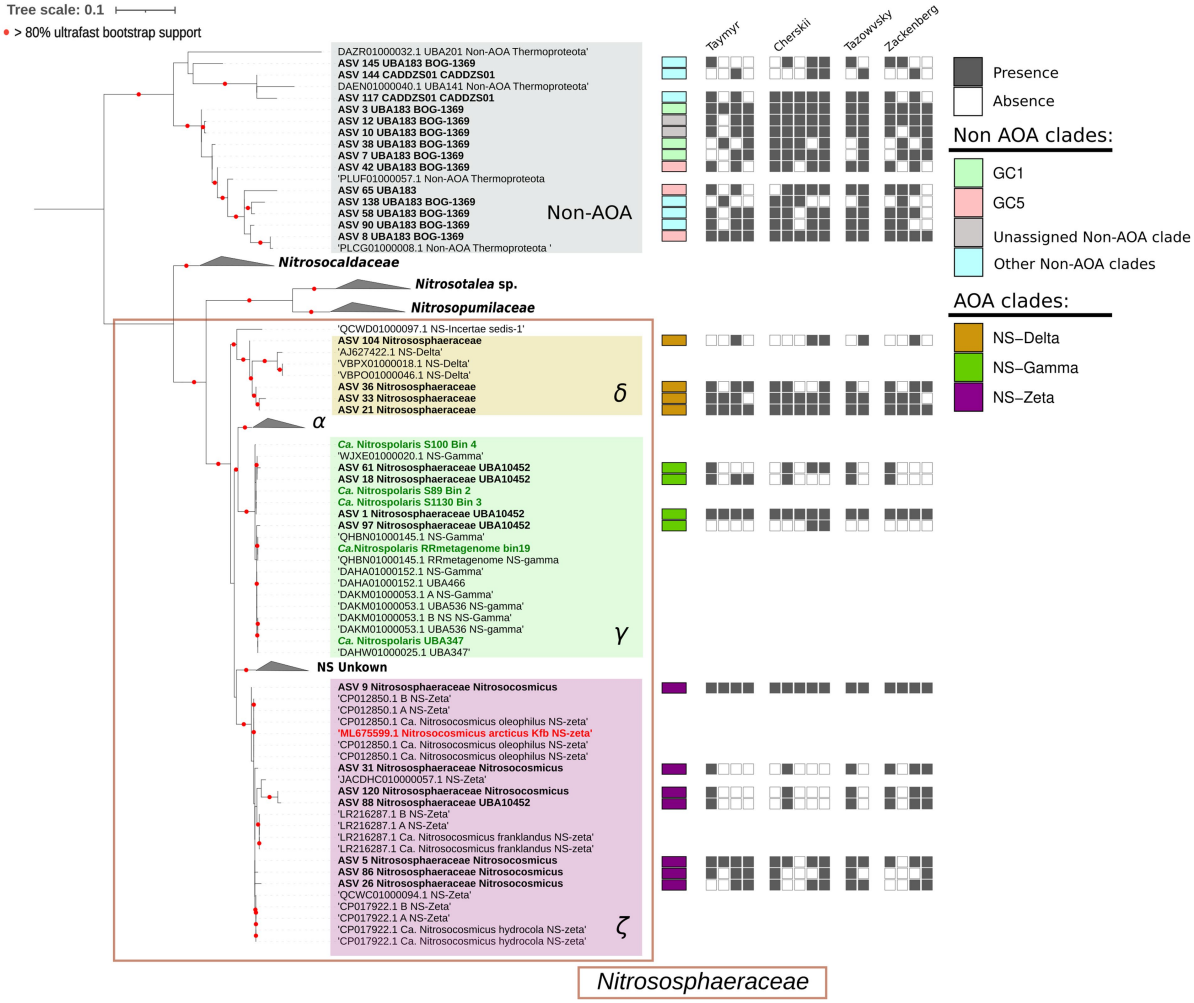
Maximum-likelihood phylogenetic reconstruction of 16S rRNA gene sequences from *Nitrososphaerales*. ASVs from this study are indicated in bold. The classification of the ASVs into *amoA* clades additionally carried out using the 16S-*amoA* correspondence database by Wang et al. (2021) is indicated by the colored boxes next to the leaves of the phylogenetic tree. The presence/absence patterns of the most abundant ASVs in the different sampling sites are indicated by the gray and white boxes, respectively. The relative abundance values of the ASVs are provided in Supplementary Table S4.

We selected the 169 ASVs corresponding to the order *Nitrososphaerales* and assigned them to *amoA* gene clades (Alves et al., 2018) using a 16S-*amoA* correspondence database (see Materials and Methods). As a result, 64 ASVs were classified into one of the following *amoA* gene clades: NS-zeta, NS-gamma, NS-delta and NS-alpha (Supplementary Figure S3). We validated this result by reconstructing a 16S rRNA gene-based maximum likelihood phylogeny using the 29 ASVs that comprised > 90% of the total relative abundance of *Nitrososphaerales* across samples (Supplementary Table S4), including also full gene sequences from AOA representing all known clades (Figure 1). Of the 169 *Nitrososphaerales* ASVs, 105 remained unclassified by the 16S-*amoA* database. We performed an extended phylogenetic reconstruction which revealed that 71 of the unclassified ASVs were clustering with the non-ammonia oxidizing *Nitrososphaerales* (non-AOA), branching as a sister group to the *Nitrososophaeraceae* family (Supplementary Figure S3). Of these, most of the ASVs sequences belonged to sublineages of the groups 1.1c (*Ca.* Gagatemarchaeaceae) and 1.3 (Supplementary Figure S3) (Biggs-Weber et al., 2020; Sheridan et al., 2023). Both the AOA and non-AOA communities exhibited low diversity, with a reduced number of ASVs detected in high relative abundances (Figure 1). In most cases, these dominant ASVs were detected across all the different sites and locations (i.e., ASV 9 NS-zeta, ASV 1 NS-gamma).

**Figure 2:**
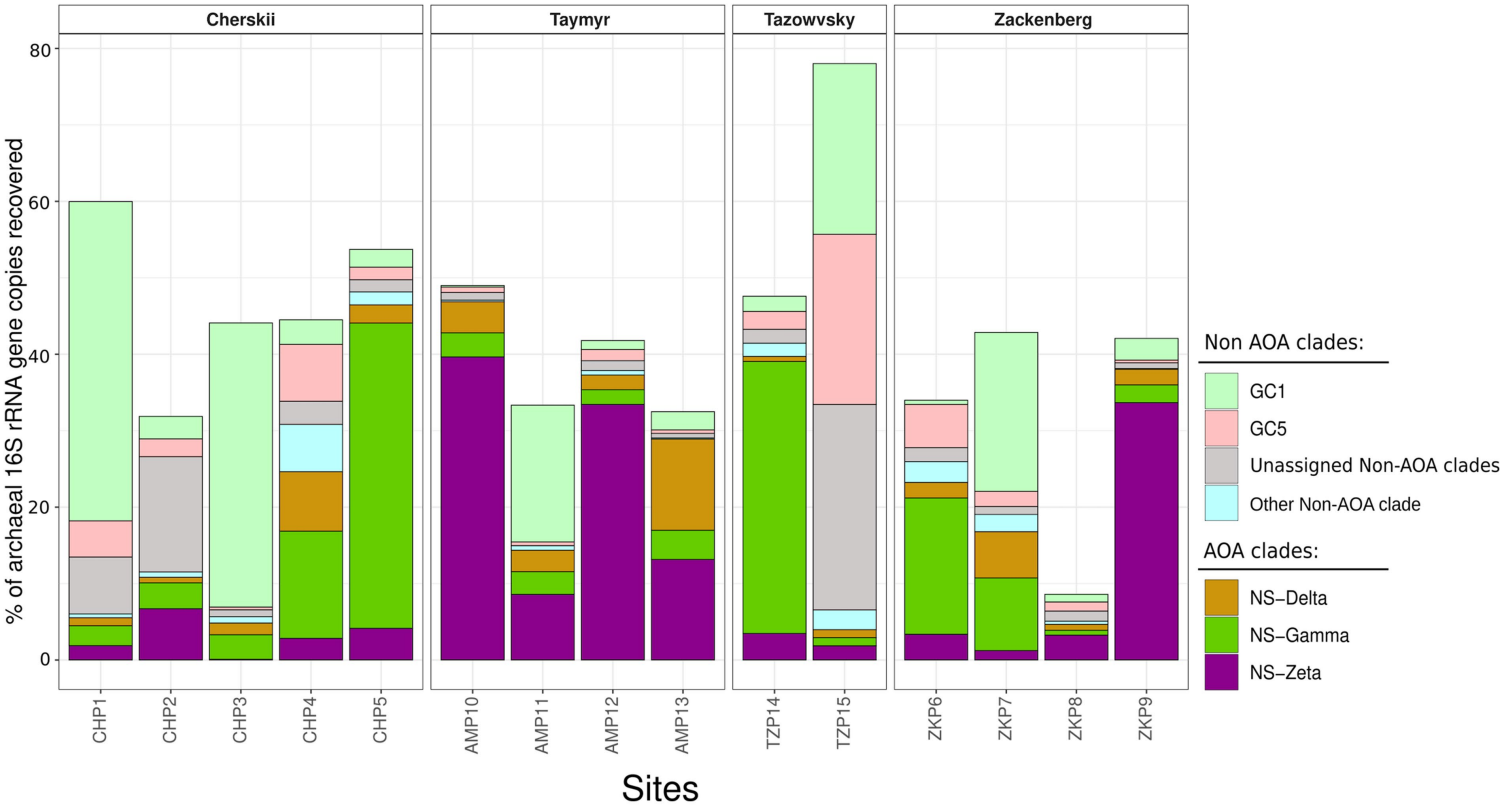
Relative abundance of AOA and non-AOA clades in the sampled circumpolar Arctic locations. Barplots show the relative abundances of all ASVs classified as *Nitrososphaerales*. The classification of AOA into the different *amoA* clades was obtained by the 16S-*amoA* database (Wang et al., 2021), and the non-AOA classification was obtained by phylogenetic placement (Supplementary Figure S2). Reads were normalized to the total count of archaeal reads.

The distribution and relative abundance of *Nitrososphaerales* ASVs varied across sites, with AOA lineages ranging from 5 to 48% and non-AOA lineages 2–71% of archaeal reads, respectively (Figure 2). AOA comprised the majority of *Nitrososphaerales’* reads (≥ 50%) at seven sites (CHP5, AMP10, AMP12, AMP13, TZP14, ZKP6, ZKP9), while non-AOA comprised the majority at four sites (CHP1, CHP2, CHP3, TZP15). The four remaining sites exhibited an almost equal relative abundance of the two groups (CHP4, AMP11, ZKP7, ZKP8). The AOA population in all sites comprised the clades NS-zeta (0.1–40% of total archaeal reads) and NS-gamma (0.7–40%), with a lower relative abundance of NS-delta (0.6–12%). Although all three clades co-occur in all sites, in most sites one clade predominated, comprising >50% of AOA reads. The non-AOA population mostly consisted of the sub- lineage 1.1c-GC1 in all sites (1.5–48%), although the other lineages were present in most sites at a lower proportion.

**Figure 3:**
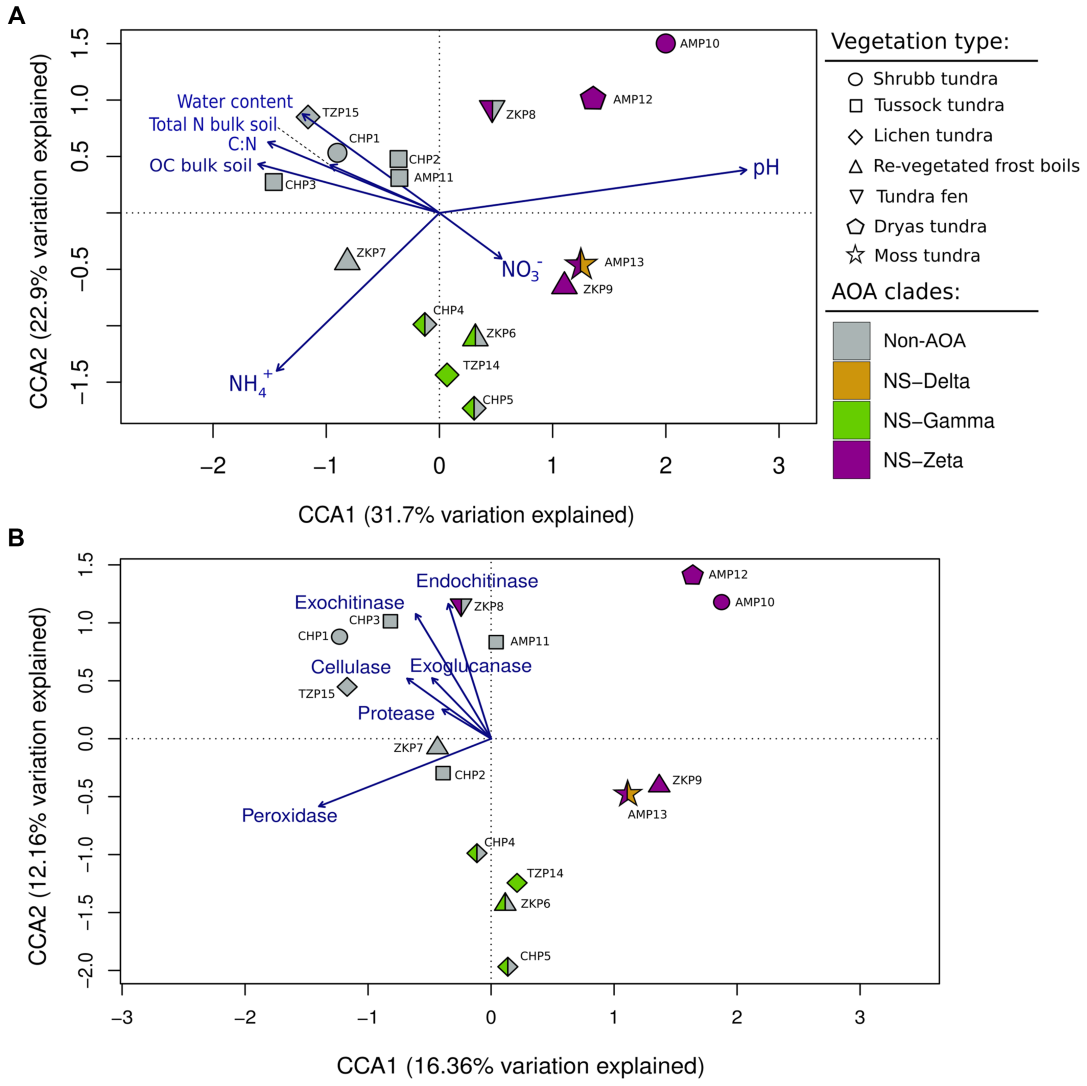
CCA biplot of the relative abundances of *Nitrososphaerales* ASVs on each sampling site and a selection of soil physico-chemical properties **(A)** and enzymatic activities **(B)**. The environmental parameters are represented by the blue arrows. Symbols represent the main vegetation type on each site. Colors represent the most abundant *amoA* clade found on each site.

The composition of *Nitrososphaerales* varied across sites within the same location, indicating that the specific characteristics of each site (e.g., vegetation, soil physicochemical properties, etc.) might be more influential to the community structure than the geographic location. To further explore the relationship between the environmental variables that could be extracted from the original studies and the *Nitrososphaerales* community composition, we built two CCA models with selected soil physicochemical parameters (A) and soil enzymatic activities (B), respectively, and the read counts data of AOA and non-AOA ASVs (Figure 3). The resulting models (A: R^2^ = 0.63, *p* < 0.05; B: R^2^ = 0.52, *p* > 0.05) show that there is a similar positioning in the ordination of sites sharing the dominant *Nitrososphaerales* clades (i.e., more than 50% of *Nitrososphaerales* reads assigned to NS-zeta, NS-gamma, NS-delta or non-AOA). Overall, in model A (Figure 3A) non-AOA and AOA separate across CCA1, indicating that non-AOA are more abundant in soils with a lower pH, higher water content as well as higher organic carbon (OC) and ammonia/total nitrogen content. On the other hand, AOA of the NS-zeta clade seem to occur more frequently in less acidic and neutral soils, while the NS-gamma clade is associated with more mineral soils with higher nitrate content. Therefore, we infer that the three major clades detected in arctic tundra soils show distinct patterns of distribution based on the available soil physicochemical parameters. Additionally, we observed the association of certain vegetation types with specific clades, as is the case for NS-gamma which is the most abundant AOA clade in three out of four lichen tundra sites, and non-AOA which are the most abundant *Nitrososphaerales* in all the tussock tundra sites. Model B (Figure 3B) illustrates that non-AOA seem to be more abundant in sites where measured activities of soil catabolic enzymes were higher. NS-zeta- and NS-gamma-dominated sites also separate from each other across CCA2, with NS-Gamma being more frequent in soils with lower enzymatic activities than NS-zeta.

### Isolation of an NS-zeta clade AOA, *Ca.* Nitrosocosmicus arcticus

In order to shed light on the ecophysiological characteristics of AOA in the arctic and understand their contribution to GHG emissions, soil samples were collected in 2009 from Svalbard (Alves et al., 2013) and used to establish enrichment cultures for AOA isolation. As a result, *Ca.* N. arcticus was successfully enriched to 93% after 7 years of continuous cultivation (Alves et al., 2019). The reported ammonia oxidation rates were exceptionally slow, with 0.5 mM NH_3_ being oxidized over 70 days. Attempts to eliminate the remaining bacterial contaminants using multiple antibiotics proved unsuccessful (Alves et al., 2019). Since then, multiple approaches were tested without success, including dilution to extinction, fluorescence-activated cell sorting (FACS) based on the autofluorescence of cofactor F_420_ encoded by AOA (Spang et al., 2012) (Supplementary Figure S4), filtration and plating utilizing the liquid–solid method (Klein et al., 2022).

Microscopic assessments of the enrichment cultures revealed that most cells sedimented at the bottom of the growth containers in the form of large aggregates visible even to the naked eye (Supplementary Figure S4). The remaining contaminants were in close association to these aggregates and likely also embedded within (Supplementary Figure S5). The application of an antibiotic mix consisting of two antibiotics known to impede or prevent biofilm formation and penetrate the biofilm matrix of bacteria, azithromycin and ciprofloxacin (Liu et al., 2019), eliminated the remaining contaminants in two to four passages. Even though the size of aggregates was drastically reduced in the presence of antibiotics, the growth of *Ca.* N. arcticus was not affected based on nitrite production.

### Nitrite production of *Ca.* N. arcticus is not stable at temperatures above 20 °C

**Figure 4:**
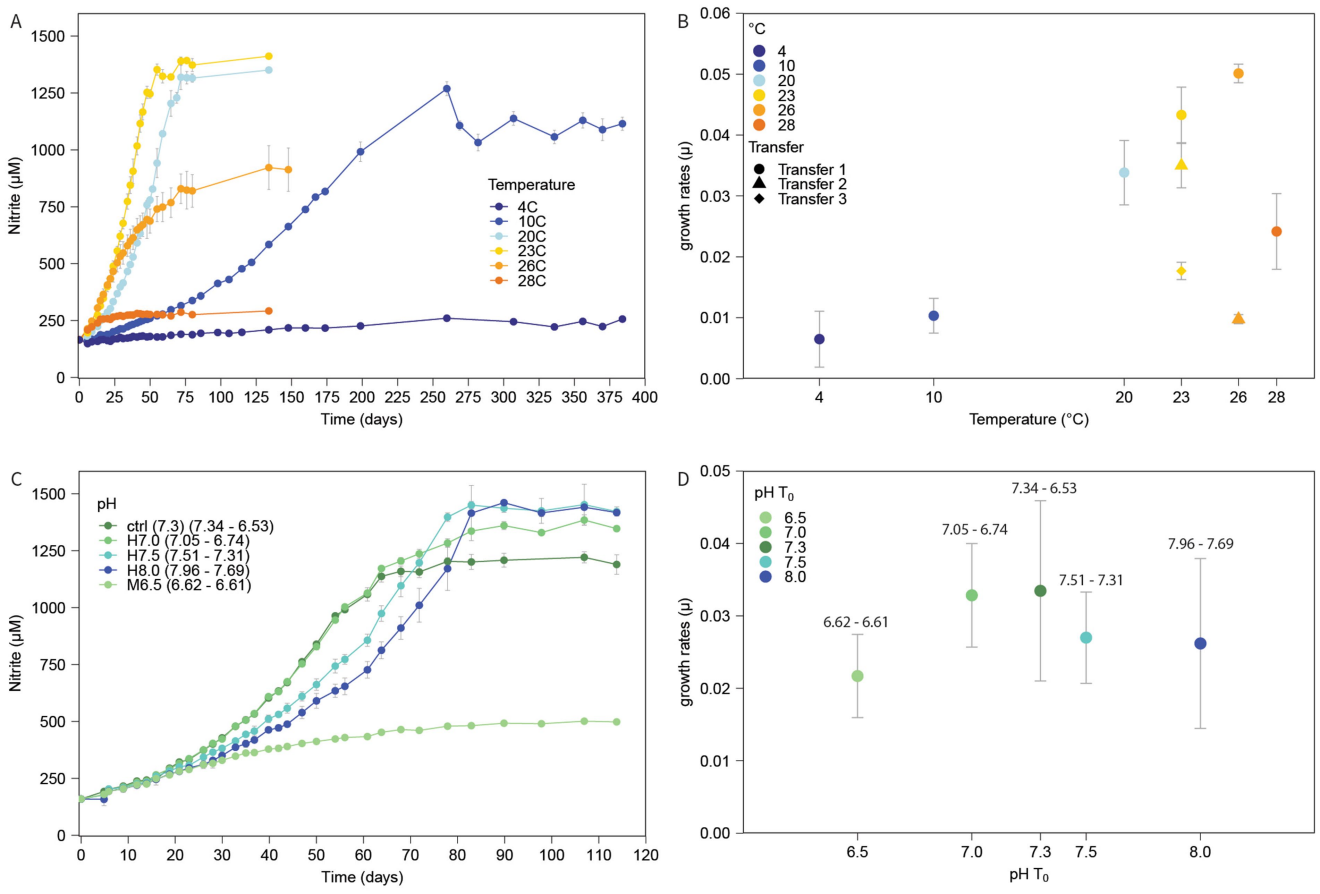
Temperature and pH range of *Ca.* N. arcticus. Nitrite production **(A,C)** and corresponding growth rates **(B,D)** of *Ca.* N. arcticus grown at different temperatures and pHs. **(A)** Growth of *Ca.* N. arcticus at temperatures between 4 °C (dark blue) to 28 °C (dark orange). Temperatures are indicated by a color scale from blue to orange. **(B)** Growth rates of each temperature. Additional transfers at the same temperature are indicated by triangles (2nd) and a diamond (3rd). **(C)** Growth of *Ca.* N. arcticus at pHs between 6.5 (light green) to 8.0 (blue). pHs are indicated by a color scale from light green to blue. MOPS and HEPES buffers were used for pHs 6.5 and 7.0, 7.5, 8.0, respectively. The control was minimally buffered by sodium bicarbonate (NaHCO_3_) in the growth medium. **(D)** Growth rates at each pH. Starting and final pHs are shown above. Growth rates were calculated based on exponential segments, or linear portions after log semi-log plotting growth data. Nitrite measurements and growth rates show averages of three biological replicates. Error bars depict the standard deviation.

The temperature in Ny-Ålesund close to the original sampling site ranged from −12 °C in winter to 3.8 °C in summer with an annual average of −5.2 °C during 1981–2010 (Førland et al., 2011). This is in stark contrast to 20 °C used for routine cultivation (Alves et al., 2019). The enrichment of *Ca.* N. arcticus was previously reported to optimally oxidize ammonia between 20 and 28 °C, while minimal or no production of nitrite was seen at 8 °C and 4 °C, respectively (Alves et al., 2019). The effect of temperatures ranging from 4 to 28 °C was reassessed using the now pure culture of *Ca.* N. arcticus over a period of more than a year (Figures 4A,B). Active nitrite production (NO_2_^prod^) was measured at all temperatures, but was not stable above 20 °C.

At 23 °C, the NO_2_^prod^ of *Ca.* N. arcticus was fastest during the initial transfer. Both the time till inhibition of ammonia oxidation and the maximum nitrite concentrations reached decreased with increasing temperatures >23 °C. Ammonia oxidation at temperatures below 20 °C was drastically reduced. It took ~260 days for all ammonium to be oxidized at 10 °C after which nitrite concentrations continued to fluctuate until stabilizing at ~1,100 μM after ~356 days. Active ammonia oxidation at 4 °C was by far the slowest, producing ~110 μM nitrite over ~260 days, after which nitrite concentrations also continued to fluctuate.

To test the effect of continued elevated temperature on *Ca.* N. arcticus, cultures grown at 23 °C and 26 °C were continuously transferred. Continued exposure to elevated temperatures strongly affected growth, decreasing observed growth rates with each consecutive transfer at 23 °C and nearly completely inhibiting growth after one transfer at 26 °C (Figures 4A,B).

### *Ca.* N. arcticus tolerates alkaline conditions

In correspondence with the increased abundance of the NS-zeta clade in less acidic to neutral soils (Figure 3), *Ca.* N. arcticus was isolated from slightly alkaline soil with a measured pH of 7.6 (Alves et al., 2013). The enrichment of *Ca.* N. arcticus was reported to actively oxidize ammonia at starting pH values between 6.0 and 8.0 when grown with urea (Alves et al., 2019). The effect of pH on the pure culture of *Ca.* N. arcticus grown with ammonia was reassessed using buffered media (“Materials and methods”) ranging from pH 6.5–8.0 (Figures 4C,D).

NO_2_^prod^ was measured at all tested pH values, with an optimum between 7.0–7.3 according to growth rate maxima (Figures 4C,D). At pH ≥ 7.5, NO_2_^prod^ was slower, but reached higher final NO_2_^−^ concentrations, whereas at pH 6.5 NO_2_^prod^ gradually decreased, reaching a maximum of only ~500 μM NO_2_^−^.

### Aggregates of *Ca.* Nitrosocosmicus arcticus display biofilm-like features

**Figure 5:**
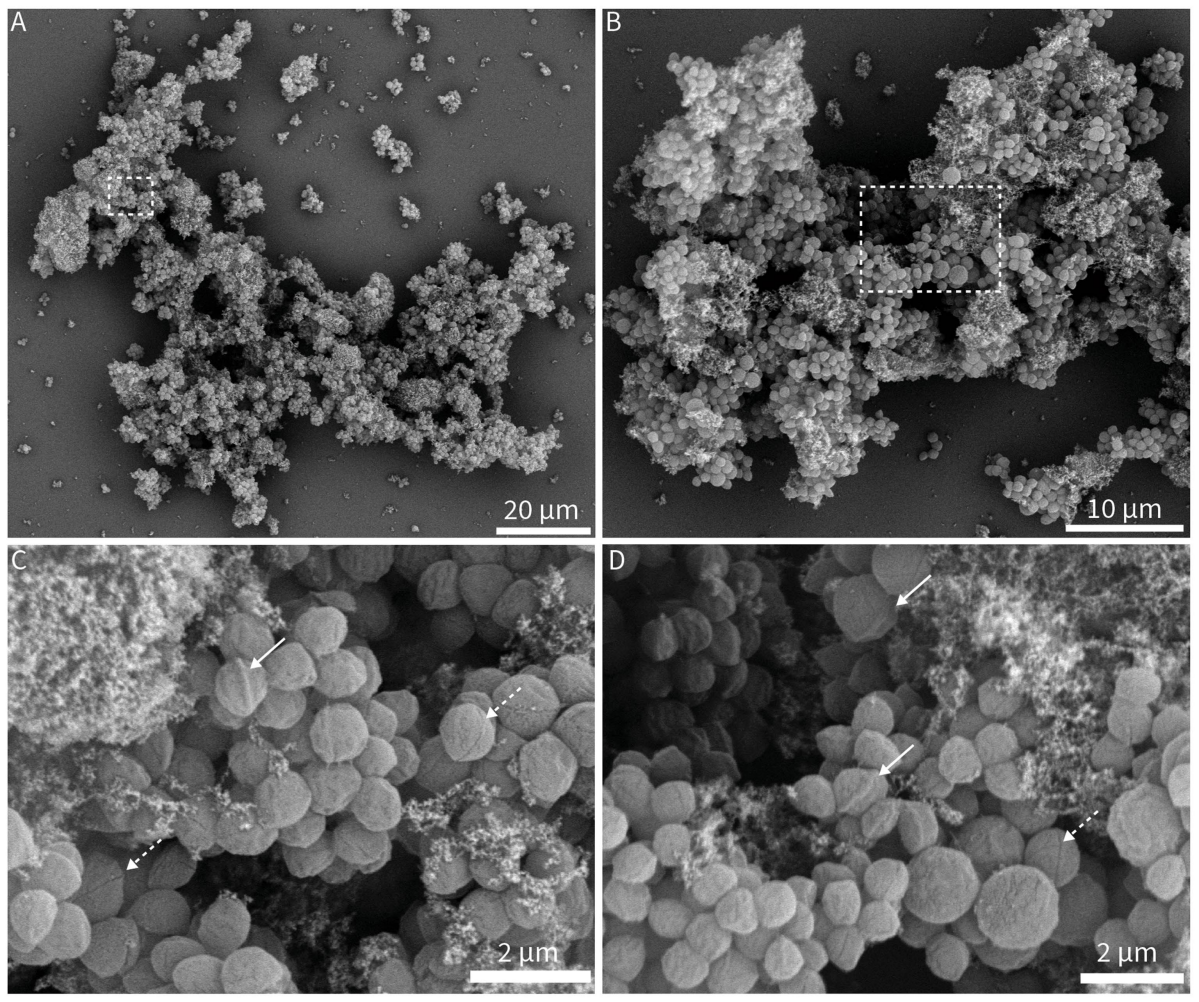
Visualization of aggregates, single cells, and putative extracellular polymeric substances (EPS) of *Ca. Nitrosocosmicus arcticus* via scanning electron microscopy (SEM). Aggregates **(A,B)** and corresponding close-ups **(C,D)** of *Ca.* N. arcticus. Putative EPS is visible as connected granular structures in between or covering cells **(C,D)**, spread throughout aggregates **(A,B)**. Cells displayed protruding (**C,D**, white arrow) or intended (**C,D**, white dashed arrow) ring-like structures along their central axes. Micrographs C and D are enlargements of areas marked with white dashed boxes in **(A,B)**, respectively.

The aggregation of multiple cells has emerged as a hallmark feature of all cultivated *Ca.* Nitrosocosmicus sp. species. Even when cultivated in liquid medium, cells are frequently observed as aggregates of varying sizes (Jung et al., 2016; Lehtovirta-Morley et al., 2016; Sauder et al., 2017; Liu et al., 2019; Alves et al., 2019). Imaging axenic *Ca.* N. arcticus cultures confirmed the extensive size of formed aggregates seen in enrichment cultures. Aggregates reached up to 100 μm in length, containing hundreds to perhaps thousands of cells (Figure 5; Supplementary Figure S4). A large proportion of the aggregates consisted of putative extracellular polymeric substances (EPS), visible as granular structures distributed throughout the aggregates or covering cells (Figure 5). The coccoid cells occasionally displayed either “walnut-like” or “yoyo-like” features, visible as a protruding or indented ring along their central axis, respectively (Figure 5).

### Unique genomic features of arctic AOA

The isolation of *Ca.* N. arcticus in pure culture followed the optimization of a protocol for high MW DNA extraction and genome closure through long-read sequencing. The resulting genome exhibits the typical characteristics of other genomes of the genus *Ca.* Nitrosocosmicus (Table 1). With a size of 2.67 Mb it is the smallest genome among *Ca.* Nitrosocosmicus sp., which in general have the largest genome sizes compared to other AOA. It harbors 3 copies of the 16S & 23S rRNA operons, single copies of the ammonia monooxygenase genes (*amoABXYZ*) and two amoC homologs, one copy of the low affinity ammonium transport protein (Amt2), and a complete set of urease genes as well as two urea transport proteins (a urea transporter family protein, TCDB 1. A.28, and a sodium symporter family protein, TCDB 2. A.21, predicted to transport urea).

**Table 1:** Genomic and physiological features of Nitrosocosmicus arcticus Kfb and other representative terrestrial AOA.

Organism	Status	Growth temp optimum (oC)	pH optimum	Genome size (Mb)	Complete-ness %	Redundancy %	No. of contigs	DNA GC %	16S/23S/5S rRNA genes	Amo A/B/C/D/X/Y/Z	Amt no/type	Urea transport UT/SSS	Ure ABC	References
*Ca.* Nitrosocosmicus arcticus Kfb	Pure	20	7.0–7.3	2.67	96.1	1.94	1	34.09	3/3/1	1/1/1/1/1/1	1/Amt2	1/1	1/1/1	This study
*Ca.* Nitrosocosmicus oleophilus MY3	Pure	30	6.5–7	3.43	98.05	0.97	1	34.14	3/3/1	1/1/3/1/1/1	1/Amt2	1/1	2/1/1	Jung et al. (2016)
*Ca.* Nitrosocosmicus franklandianus C13	Pure	40	7	2.84	98.05	1.94	1	34.07	2/2/1	1/1/3/1/1/1	1/Amt2	1/1	1/1/1	Lehtovirta-Morley et al. (2016)
*Ca.* Nitrosocosmicus hydrocola G61	Enriched	33	8	2.99	99	2.91	1	33.94	2/2/1	1/1/2/1/1/1	1/Amt2	1/1	1/1/1	Sauder et al. (2017)
*Ca.* Nitrosocosmicus agrestis SS	Enriched	37	6.5–7	3.22	96.1	2.91	43	33.42	2/2/1	1/1/3/1/1/1	1/Amt2	1/1	1/1/1	Liu et al. (2021)
*Ca.* Nitrosopolaris wilkensis RR	–	–	–	3.99	90.8	3.9	278	38.6	1/1/1	1/1/−/1/1/1	2/Amt1, Amt2	1/−	1/1/1	Pessi et al. (2022)

As the main metabolic features of *Ca.* N. arcticus were discussed before (Alves et al., 2019), we sought to identify potential adaptations shared by the only available AOA genomes from arctic environments, namely *Ca.* N. arcticus and *Ca.* Nitrosopolaris MAGs. Our comparative genomics approach identified 11 orthologous protein families shared exclusively by *Ca.* N. arcticus and one or both of the polar genomes of *Ca.* N. wilkensis and *Ca.* N. rasttigaisensis (Supplementary Table S5). Among the protein families for which a functional annotation could be provided, we find protein families putatively involved in nucleic acid interactions such as a DNA primase/polymerase family protein typically involved in replication and repair (Guilliam et al., 2015), a HEPN-domain containing protein, that typically act as endo-RNAses in toxin-antitoxin systems (Anantharaman et al., 2013) and a RatA family toxin which can bind to and inhibit the association of ribosome subunits (Bertram and Schuster, 2014). Moreover, the arctic genomes share two protein families that might be involved in membrane-related functions: a KAP-family NTase, typically involved in regulating the assembly of membrane-associated protein complexes (Aravind et al., 2004), and a SAM-dependent methyltransferase with weak identity hits to menaquinone biosynthesis methyltransferases. *Ca.* N. arcticus-specific protein families (8 orthogroups and 62 genes not assigned to an orthogroup; Supplementary Table S6) include a Taurine Uptake Transporter (TauT) Family protein, two Cation: Proton Antiporter (CPA2) Family proteins with close similarity to Na^+^/H^+^-K^+^ antiporters, two glucose/sorbosone family dehydrogenases, two putative adhesion-related proteins and a MEDS domain-containing protein, the latter putatively involved in sensing hydrocarbon derivatives (Anantharaman and Aravind, 2005).

## Discussion

### Specific clades of *Nitrososphaerales* are involved in nitrogen and carbon cycling in arctic tundra soils

The archaeal populations in the circumpolar arctic, which our study targeted, comprise mainly representatives of *Thermoproteota* (specifically AOA) and *Methanobacteriota*, two lineages responsible for the production of the GHG gasses N_2_O and CH_4_, respectively, through direct and indirect pathways. As the role of *Methanobacteriota* in the context of a warming arctic has been extensively studied (e.g., Wang et al., 2025), we focus here on investigating the diversity and distribution of *Thermoproteota*, which in this case comprise the class *Nitrososphaeria*, order *Nitrososphaerales*.

In contrast to previous studies which focused on sequencing the *amoA* gene, our archaea-targeted 16S rRNA approach allowed for the untargeted characterization of the *Nitrososphaerales* community. In line with previous studies (Alves et al., 2013; Siljanen et al., 2019), our results corroborated that the diversity of AOA communities is very low across all sites, as it comprised few phylotypes of the clades NS-gamma, NS-delta and NS-zeta (Alves et al., 2018). However, our approach also revealed the importance of the non-AOA *Nitrososphaerales* of the recently described family of *Ca.* Gagatemarchaeaceae (formerly known as group 1.1c) and group 1.3 (Biggs-Weber et al., 2020; Sheridan et al., 2023). These lineages were also detected in all investigated sites, and exhibited low diversity with the majority of phylotypes belonging to groups GC1 and GC5 within the recently described candidate genus *Ca.* Gagatemarchaeum (Sheridan et al., 2023).

The majority of phylotypes were detected in all sampled locations across the circumpolar Arctic spanning very large distances, indicating that the climatic and geochemical characteristics of arctic tundra soils selectively impact *Nitrososphaerales*, as observed before (Alves et al., 2013). This reflects the increased selection pressures seen in all extreme environments (Mao and Grogan, 2012), with presumably the particular temperature conditions being among the determining factors here. However, the three major lineages show clearly distinct and mutually exclusive abundance patterns, and although geographic location is not a determining factor in their distribution, parameters such as pH, concentrations of ammonium, nitrate, organic carbon, total nitrogen and water content are shown to selectively influence the distribution of *Nitrososphaerales* in our study, in accordance with previous studies (Zhalnina et al., 2012).

Non-AOA lineages exhibited the highest relative abundance in sites richer in organic carbon and nitrogen, with higher water content and lower pH, which expectedly displayed also higher activities of enzymes associated with organic matter degradation, such as all the tussock tundra sites. Available genomic information of the 1.1c lineages associated with the genus *Ca.* Gagatemarchaeum, where the most abundant non-AOA ASVs in our samples clustered, suggests that most are aerobic heterotrophs, and as such would benefit from the variety in available sources of organic carbon released by catabolic enzymatic activities, for which genes are also found in these genomes (Sheridan et al., 2023). Additionally, they encode adaptations involved in acid tolerance such as the V-type ATPase (Sheridan et al., 2023) and are generally acidophilic (Oton et al., 2016). Finally, they are abundant in sites with high water content indicating a possible microaerophilic lifestyle (Biggs-Weber et al., 2020; Oton et al., 2016).

The AOA lineages NS-gamma and NS-zeta exhibited an inverse relative abundance pattern in our samples, underscoring their distinct ecophysiological capabilities and niche specialization. The highest relative abundance of the NS-zeta clade was observed in less acidic to neutral soils (5.25 ± 0.14–6.5 ± 0.57), with pH being a clear driver of niche separation between the two clades (Figure 3). This is congruent with soil pH being continuously reported as an important factor in the niche separation of terrestrial AOA (Alves et al., 2018; Gubry-Rangin et al., 2011; Nicol et al., 2008). This was also the most abundant AOA clade in sites with high carbon-to-nitrogen (C: N) ratios and high OC, typical of tundra fen environments. The fact that in our CCA model organic matter concentration does not seem to affect the distribution of this clade is rather surprising, as most isolates of the genus have been isolated from rather nutrient rich environments with relatively high OC values or a constant influx of OC compounds (Jung et al., 2016), and are also abundant in arctic peatlands (Siljanen et al., 2019). Additionally, it has been shown that NS-zeta isolates have the lowest affinity to ammonia among AOA (Jung et al., 2022), potentially explaining its frequent presence in nutrient-rich environments, such as fertilized soils, peatlands and wastewater treatment plants (Liu et al., 2021; Mußmann et al., 2011). Conversely, NS-gamma AOA exhibited the highest relative abundance in organic-poor samples with low C: N ratios and the lowest OC and total N concentrations among all analysed samples, indicative of erosion-prone and well-drained conditions of lichen tundra vegetation, as well as revegetated frost boils. Their distribution is inversely associated with pH and OC and partially associated to nitrate concentration in our CCA model. This is in line with the reported distribution of this lineage in acidic nutrient-poor mineral soils from polar regions (Pessi et al., 2022).

We acknowledge that due to the limitations of the CCA analysis, we can only infer indirect associations between the analyzed variables and the microbial populations. Since the number of explanatory variables that could be extracted from the original studies and included in the models is limited and they often describe complex environmental parameters, these associations should be interpreted with caution. Overall, this analysis provides strong evidence that the three microbial populations occupy distinct ecological niches in the Arctic.

### Temperature range of *Ca.* Nitrosocosmicus arcticus reveals vulnerability of nitrification in a warming arctic

The only available AOA strain from an arctic environment, *Ca.* N. arcticus, was initially enriched from an arctic tundra frost boil in Svalbard (Alves et al., 2019) and its isolation into a pure culture is described in this study. The sample isolation site is typical of the distribution parameters we identified in this study for the NS-zeta clade, and as such it can be considered a representative arctic NS-zeta clade AOA. Thus, its physiological characterisation and exploration of the conditions that can limit its activity and growth can offer insights on the environmental conditions that could affect nitrification in a rapidly changing arctic environment.

Active ammonia oxidation was observed at all tested temperatures ranging from 4 °C to 28 °C but remained stable only until 20 °C. Prolonged exposure of *Ca.* N. arcticus to temperatures above 20 °C gradually reduced NO_2_^prod^ resulting in starkly reduced (23 °C) or complete inhibition of NO_2_^prod^ (>23 °C). Average ground temperatures in the Arctic are still far from reaching 20 °C, with annual ground surface temperatures of ~0 °C and −2.5 °C at 0 m and 5 m depth, respectively, in 2024,^1^ modelled to increase at a maximum rate of 0.083 ± 0.048 °C/year in the Svalbard archipelago (Grünberg et al., 2024). However, near-surface ground temperatures (0.03 m depth) already reach up to 10 °C in July and August (Isaksen et al., 2022) and temperatures are modelled to continuously rise in the future (Hansen et al., 2014), with the rate of warming of the Svalbard archipelago being twice the Arctic average and roughly seven times the global average since 1991 (Nordli et al., 2020). It is therefore possible for upper soil layers to reach temperatures above 20 °C in the future, especially during extreme weather events which are predicted to occur more frequently in the Arctic due to global warming (Hansen et al., 2014).

Until this tipping point is reached, rising temperatures will rather increase the activity of ammonia oxidation in arctic soils, as the growth and therefore NO_2_^prod^ of *Ca.* N. arcticus is fastest at temperatures well above arctic *in-situ* conditions. In turn this could lead to a positive feedback loop for global warming as AOA-mediated nitrification was reported to be a key driver in high N_2_O emission from arctic peatland soils both indirectly by being the main source of NO_2_^−^ in arctic soils, fueling N_2_O production via denitrification and directly by producing N_2_O as a byproduct of ammonia oxidation (Supplementary Figure S6) (Siljanen et al., 2019). This would further exacerbate atmospheric N_2_O levels which have increased by almost 25% from pre-industrial times in 1725 to 2022, reaching levels higher than at any time during the last 800,000 years with increase rates in the 20th century being the highest over the past 20,000 years (Tian et al., 2024).

### Nitrogen source utilization and biofilm forming potential might expand the pH range of *Ca.* N. arcticus

The ability of *Ca.* N. arcticus to oxidize ammonia across a broad pH range (6.5–8.0) indicates a physiological flexibility that is notable given the occurrence of this clade (NS-zeta) in arctic soils of even lower pH values. While this range is congruent with that previously reported for the enrichment culture of *Ca.* N. arcticus (pH 6.0–8.0) (Alves et al., 2019) the shift in optimum pH from 6.0 in the enrichment to 7.0–7.3 in the pure culture suggests that experimental design and environmental context strongly influence apparent pH preferences. A key difference between these studies is the nitrogen source used: urea in the *Ca.* N. arcticus enrichment experiments (Alves et al., 2019) versus ammonium chloride in all other enriched or isolated *Nitrosocosmicus* species (Jung et al., 2016; Lehtovirta-Morley et al., 2016; Liu et al., 2019; Sauder et al., 2017, this study: https://www.zotero.org/google-docs/?2S1UId). Urea hydrolysis is known to increase a solution’s pH, and since only initial pH values were measured in the enrichment experiments, it is possible that microbial urea degradation could have elevated the pH of the unbuffered medium, in turn alleviating inhibitory effects on ammonia oxidation and obscuring the true lower pH limit of *Ca.* N. arcticus activity.

These observations underscore the importance of nitrogen source utilization in shaping the pH niche of AOA. Although several AOA lineages are well adapted to acidic, ammonia-limited soils (Alves et al., 2018; Gubry-Rangin et al., 2011; Nicol et al., 2008), it remains unresolved whether all AOA detected in such environments are obligate low-pH specialists. Instead, some AOA may rely on indirect mechanisms, such as microenvironmental buffering through the use of other substrates (e.g., urea), to persist and remain active under acidic conditions, enabling continued nitrification even at pH values as low as 4 (Burton and Prosser, 2001). For *Ca.* N. arcticus, this metabolic flexibility may be particularly relevant in arctic soils, where nutrient availability and pH can fluctuate spatially and temporally. It should be noted that growth of enrichment cultures of *Ca.* N. arcticus without detectable oxidation of ammonia was reported previously (Alves et al., 2019), but was never observed during experiments with pure cultures. This does not however exclude the possibility of an auxiliary or alternative energy metabolism indicated by the expanded repertoires of genes to use alternative substrates in all so far analyzed genomes of *Nitrosocosmicus* sp. (Alves et al., 2019).

Beyond substrate use, biofilm and aggregate formation likely represent an additional mechanism facilitating activity under suboptimal pH conditions. Biofilms have been hypothesized to enable nitrification at low pH by creating microhabitats with altered chemical conditions, including localized pH buffering (Burton and Prosser, 2001; Gubry-Rangin et al., 2011). The extensive aggregate formation observed for *Ca.* N. arcticus, with structures reaching ~100 μm in length containing thousands of cells and permeated with putative EPS (Figure 5; Supplementary Figure S4), supports this idea. Such aggregates were even visible to the naked eye prior to sample processing, suggesting that *in situ* structures may be substantially larger and more complex than those observed microscopically.

Aggregate formation appears to be a conserved trait within the *Ca.* Nitrosocosmicus clade and all isolated members have the genetic capacity for EPS production (Alves et al., 2019; Jung et al., 2016; Lehtovirta-Morley et al., 2016; Liu et al., 2019; Sauder et al., 2017). Recent evidence that diverse AOA readily form biofilms when maintained on solid surfaces further suggests that biofilm-associated growth may represent the dominant lifestyle of terrestrial AOA rather than an exception (Dreer et al., 2025). For AOA from the NS-zeta clade, such a lifestyle may have broadened their ecophysiological niche as evidenced by their distribution in soils from pH 5.25 to 6.5, despite the apparent sensitivity of the pure cultures to acidity.

In addition to pH-related effects, biofilm formation likely confers broader ecological advantages in cold and oligotrophic environments. Biofilms are known to enhance resistance to physical and chemical stresses such as desiccation, antibiotic toxicity and temperature extremes (Flemming et al., 2016). In cold environments specifically, biofilm systems can outperform suspended-growth systems, with active nitrification observed at temperatures as low as 1 °C (Ahmed et al., 2019). Many microorganisms increase biofilm thickness and/or adherence to surfaces under cold stress, potentially stabilizing metabolic activity under otherwise inhibitory conditions (Ahmed and Delatolla, 2020; Lee et al., 2017; Qiao et al., 2021; Rihab, 2012). The presence of genes involved in cell surface modification and adhesion in the shell genome of *Ca.* N. arcticus is consistent with a biofilm-mediated stress tolerance strategy, supporting the hypothesis that aggregation and EPS production play central roles in the ecological success of this organism in arctic soils.

### Genome investigations of arctic AOA offer insights into adaptation mechanisms

We attempted to uncover putative adaptation strategies of the two dominant AOA clades in the arctic ecosystem through comparative genomics, and identify the genomic repertoire that enables them to occupy distinct environmental niches. Arctic ecosystems endure particular environmental stress factors, such as low temperatures, which can cause cellular damage by affecting the permeability of the cell membrane, cause misfolding of proteins and DNA damage (Zhang and Gross, 2021); extended UV exposure during the summer months as well as nutrient supply challenges due to slow nutrient cycling (Stark, 2007). Among the orthologous protein families shared by *Ca.* N. arcticus and the *Ca.* Nitrosopolaris sp. genomes we identified a RatA family protein that could offer the ability to tightly regulate and arrest translation, potentially to optimize energy expenditure under cold stress. While this is a known function of canonical cold-shock proteins (which act on the mRNA), homologs of which exist in most AOA genomes analysed to date, this protein might offer an additional control mechanism. Furthermore, the genomes shared DNA repair proteins and chaperonins, functions that occur frequently in cold-adapted archaea (Cavicchioli, 2006).

Comparative genomics also enables us to speculate on the strategies responsible for the successful colonization of polar ecosystems by the specific AOA clades, NS-zeta and NS-gamma. Genomes from these clades share a number of genes and pathways that could enhance their survival rates in environments with fluctuating nutrient conditions as well as exposure to environmental stress in the form of temperature and UV. Firstly, they are equipped with an enzymatic repertoire that offers a high degree of versatility in terms of carbon utilisation among AOA: NS-zeta genomes are enriched in PQQ-dependent glucose/sorbosone dehydrogenase family proteins, which could supplement their electron pool via sugar oxidation (Alves et al., 2019), while *Ca.* Nitrosopolaris genomes encode a TPP-dependent alpha-keto acid decarboxylase, involved in amino acid fermentation, a branched-chain amino acid transporter system, and the glycine cleavage system, responsible for the oxidative breakdown of glycine, simultaneously releasing carbon dioxide (CO_2_) and ammonia (NH_3_). Both clades, uniquely among AOA, also encode full pathways for the biosynthesis of the molybdenum cofactor (Moco), as well as DMSO/TMAO reductase family proteins with possible metabolic or detoxification roles (Alves et al., 2013).

Regarding nitrogen metabolism, genomes from both clades encode cytochrome P450 family homologs, shown to be upregulated under acidic conditions and putatively mediating N_2_O production in *Ca.* N. oleophilus (Jung et al., 2019). *Ca.* Nitrosopolaris sp. genomes interestingly encode genes which could putatively mediate the degradation of alkylnitronates to nitrite and eventually ammonium, catalyzed by a nitronate monooxygenase and an assimilatory nitrite reductase (NiR) hemoprotein, respectively. Alkylnitronates are volatile organic nitrate compounds produced through the NOx-mediated oxidation of biogenic volatile organic compounds (BVOCs) and present in the arctic atmosphere and which could reach the soil through deposition (Andersen et al., 2025; Fischer et al., 2002). This pathway could supplement ammonium supply for ammonia oxidation in the N-limited arctic soils and perhaps play a role in adaptation to organic-depleted soils. In addition, unlike *Ca.* Nitrosocosmicus sp. genomes, *Ca.* N. wilkensis harbors two homologs of Amt-family ammonium transporter proteins of both the high (Amt1) and low affinity (Amt2) type, which would facilitate colonization of mineral soils.

Chaperone and repair functions are also enriched in both clades’ genomes (Abby et al., 2020; Pessi et al., 2022). Both encode components of the non-homologous end joining (NHEJ) DNA repair pathway, mediating the repair of double strand breaks caused by ionizing radiation and UV exposure, a crucial tool in the arctic environment.

## Conclusion

Our results corroborate previous studies indicating that nitrification in arctic tundra soils is mediated by two specific clades of AOA, NS-zeta and NS-gamma, which in turn exhibit niche specialization towards either nutrient-rich or nutrient-poor environments. In addition, for the first time our study reveals the importance of non-AOA *Nitrososphaerales* of the family *Ca.* Gagatemarchaceae and group 1.3 in arctic tundra top soils, with abundance values similar or in some cases higher than AOA. This finding underscores the role of *Nitrososphaerales* as key players in carbon cycling in arctic soils, not only as primary producers through carbon fixation but also controlling bioavailable carbon through degradation of organic carbon.

We have also isolated in pure culture the first AOA from an arctic environment, *Ca.* Nitrosocosmicus arcticus strain Kfb, belonging to the cosmopolitan, terrestrial *amoA* clade NS-zeta. It is the first described instance of an AOA able to oxidize ammonia at temperatures as low as 4 °C under laboratory conditions, and with the maximal (and optimum) temperature for stable growth at 20 °C, it represents the first psychrophilic ammonia-oxidizing archaeon. The isolation of this ammonia-oxidizing strain presents unique opportunities for future studies on nitrification in the Arctic.

## Data Availability

The datasets presented in this study can be found in online repositories. The closed genome sequence of *Ca.* N. arcticus Kfb has been deposited and is publicly available in the Microbial Genome Annotation and Analysis Platform Microscope (https://mage.genoscope.cns.fr) (Vallenet et al., 2019). The 16S rRNA amplicon sequences generated in this study are available in NCBI under BioProject PRJNA1381318.

## Footnotes

1
https://cryo.met.no/en/permafrost/bayelva
