# Supplementary material for: Environmental distribution, physiology and genomic adaptations of arctic ammonia-oxidizing archaea
Source: Front Microbiol. 2026 Mar 9;17:1722591. doi: 10.3389/fmicb.2026.1722591 (PMC13006922; doi:10.3389/fmicb.2026.1722591)
Supplement: Supplementary file 1 [file Data_Sheet_1.zip › Supplementary Material & 16S-amoA_db/Supplementary Material.docx]

# **Supplementary Material**

Environmental distribution, physiology and genomic adaptations of arctic ammonia-oxidizing archaea

Marina Montserrat Díez*^1^, Maximilian Dreer*^1^, Christa Schleper^1^ , Melina Kerou^1^

*These authors contributed equally

^1^ Department of Functional and Evolutionary Ecology, Archaea Biology and Ecogenomics Unit, University of Vienna, Djerassiplatz 1, 1030 Vienna, Austria

Correspondence addressed to: [melina.kerou@univie.ac.at](mailto:melina.kerou@univie.ac.at)

# **Supplementary Methods**

**Usage of the 16S-*amoA* database:**

The files needed to run the 16S rRNA gene-to-*amoA*-clade correspondence pipeline are:

**1_unique_16S_rRNA_gene_AOA.fasta**

contains unique (dereplicated) AOA 16S rRNA gene sequences

**2_unique_16S_amoA_taxonomy.csv**

contains the *amoA* gene clade annotations for the corresponding 16S rRNA genes, based on the *amoA* taxonomy proposed by Alves et al., 2019.

This code can be used to obtain the *amoA* gene clade taxonomy from 16S rRNA gene sequences:

makeblastdb -dbtype nucl -in 1_unique_16S_rRNA_gene_AOA.fasta -out 16S_amoA_blast_db

blastn -query 16S_seq_AOA.fasta -db 16S_amoA_blast_db -out blastn -outfmt "7 std qseqid qacc sallseqid" -max_target_seqs 3

#Eliminate from blast output all the description lines (with a # in front)

grep -v '^#' blastn_output.txt > filtered_output.txt

#Merge blast output with mapping file with the clade info:

LC_ALL=C join -a1 -1 2 -2 1 -e'-' -t $'\t' -o1.1,0,1.3,1.11,2.2 <(LC_ALL=C sort -k2 filtered_blastn.txt) <(LC_ALL=C sort -k1 2_unique_16S_amoA_taxonomy.txt) | LC_ALL=C sort k1 > blast_hits_AOA_clades.txt

# **Supplementary Tables and Figures**

**Table S1**: Samples descriptions for site, soil horizons, vegetation types and original sample pooling

| **Sample name** | **Location** | **Description site** | **Vegetation type** | **Horizon layer** | **# of samples in the pool** | **Original samples** | **References** |
| --- | --- | --- | --- | --- | --- | --- | --- |
| CHP0 | Cherskii | shrubby grass tundra | Betula exilis, Salix phenophylla, Carex lugens, Calamagrostis holmii, Aulacomnium turgidum | Ajj | 1 | CHA2 | Gittel et al., 2014a |
| CHP1 | Cherskii | shrubby grass tundra | Betula exilis, Salix phenophylla, Carex lugens, Calamagrostis holmii, Aulacomnium turgidum | O | 3 | CHA3, CHB7, CHC16 | Gittel et al., 2014a |
| CHP2 | Cherskii | shrubby tussock tundra | Eriophorum vaginatum, Carex lugens, Betula exilis, Salix pulchra, Aulacomnium turgidum | O | 6 | CHD9, CHD10, CHE7, CHE10, CHF5, CHF6, | Gittel et al., 2014a |
| CHP3 | Cherskii | shrubby tussock tundra | Eriophorum vaginatum, Carex lugens, Betula exilis, Salix pulchra, Aulacomnium turgidum | O | 3 | CHG11, CHH6, CHI12 | Gittel et al., 2014a |
| CHP4 | Cherskii | Shrubby lichen tundra | Betula exilis, Vaccinium uligonosum, Flavocetraria nivalis, Flavocetraria cucullata | A | 1 | CHH7 | Gittel et al., 2014a |
| CHP5 | Cherskii | Shrubby lichen tundra | Betula exilis, Vaccinium uligonosum, Flavocetraria nivalis, Flavocetraria cucullata | Ajj | 1 | CHI4 | Gittel et al., 2014a |
| ZKP6 | Zackenberg | Tundra vegetation with old frost boils, (already re-vegetated by lichens and some higher plants, e.g. Dryas octopetala) and some frost cracks | Vaccinium uliginosum, Salix arctica, Carex sp., Polygonum viviparum, Dryas octopetala,mosses, lichens on bare soil patches | O | 3 | ZKA1, ZKB1, ZKC1 | Gittel et al., 2014b |
| ZKP7 | Zackenberg | Tundra vegetation with old frost boils, (already re-vegetated by lichens and some higher plants, e.g. Dryas octopetala) and some frost cracks | Vaccinium uliginosum, Salix arctica, Carex sp., Polygonum viviparum, Dryas octopetala,mosses, lichens on bare soil patches | Ajj “young” | 1 | ZKB4 | Gittel et al., 2014b |
| ZKP8 | Zackenberg | Wet fen with ~ 5 % mossy hummocks on flat surface | Carex species, Eriophorum angustifolium (~5%), mosses, several grasses | O | 3 | ZKD1, ZKE1, ZKF1 | Gittel et al., 2014b |
| ZKP9 | Zackenberg | Almost flat site with active frost boils and earth hummocks (differences in relief up to 30 cm) | Cassiope tetragona, Salix arctica, Vaccinium uliginosum, Dryas octopetala, grasses, Carex sp., mosses; some lichens on bare soil patches | O | 2 | ZKG1, ZKH1 | Gittel et al., 2014b |
| AMP10 | Taymyr | shrubby grass tundra | Betula nana, Dryas punctata, Vaccinium uliginosum, Carex arctisibirica, Aulacomnium turgidum | O | 3 | AMA10, AMB10, AMC11 | Gentsch et al., 2015 |
| AMP11 | Taymyr | shrubby tussock tundra | Cassiope tetragona, Carex arctisibirica, Aulacomnium turgidum | O | 3 | AMD14, AME12, AMF11 | Gentsch et al., 2015 |
| LGP12 | Taymyr | Dryas tundra | Dryas punctata, Rhytidium rugosum, Hylocomium splendends | O | 2 | LGB9, LGC17 | Gentsch et al., 2015 |
| LGP13 | Taymyr | Grassy moss tundra | Betula nana. Carex arctisibirica, Hylocomium splendens, Tomentypnum nitens | O | 2 | LGD17, LGE16 | Gentsch et al., 2015 |
| TZP14 | Tazowvsky | Shrubby lichen tundra | Empetrum nigrum, Ledum palustre, Betula nana, Cladonia rangiferina, C. stellaris | O | 3 | TZA11, TZB11, TZC12 | Gentsch et al., 2015 |
| TZP15 | Tazowvsky | larch woodland with shrubby lichen understory (forest-tundra zone) | Larix sibirica, Ledum palustre, Betula nana, Vaccinium uliginosum, Cladonia rangiferina, C. stellaris | O | 3 | TZD6, TZE7, TZF6 | Gentsch et al., 2015 |

##

## **Table S2**: Soil physicochemical parameters* from the circumpolar arctic sampling sites.

| **Sample** | **pH** | **Watercontent (fm%)** | **OC bulk soil (wt%)** | **total N bulk soil (wt%)** | **NH4+ (µg N g-1 dm)** | **NO3- (µg N g-1 dm)** | **C:N** |
| --- | --- | --- | --- | --- | --- | --- | --- |
| CHP1 | 5.17 ± 0.13 | 54.62 ± 2.26 | 23.02 ± 1.24 | 0.98 ± 0.08 | 3.27 ± 2.77 | 0.12 ± 0.18 | 23.65 ± 1.88 |
| CHP2 | 5.25 ± 0.14 | 66.59 ± 4.99 | 23.91 ± 5.46 | 1.23 ± 0.14 | 9.1275 ± 6.52 | 0.10 ± 0.2 | 19.42 ± 3.92 |
| CHP3 | 4.64 ± 0.39 | 75.05 ± 4.24 | 26.63 ± 10.36 | 0.96 ± 0.32 | 10.62 ± 9.51 | 1.72 ± 2.54 | 27.28± 3.11 |
| CHP4 | 5.26 | 29.74 | 3.89 | 0.2 | 2.7 | 0.68 | 19.45 |
| CHP5 | 5.72 | 48.42 | 6.63 | 0.42 | 6.92 | 0.7 | 15.79 |
| ZKP6 | 5.6 ± 0.1 | 51.35 ± 1.83 | 15.2 ± 0.64 | 0.99 ± 0.04 | 6.37 ± 6.5 | 0.17 ± 0.02 | 15.35± 0.24 |
| ZKP7 | 5.4 | 43.87 | 10.08 | 0.59 | 0.97 | 0.08 | 17.08 |
| ZKP8 | 5.29 ± 0 | 77.24 ± 4.19 | 28.96 ± 2.38 | 1.43 ± 0.13 | 2.13 ± 3.07 | 1.12 ± 1.15 | 20.34 ± 1.36 |
| ZKP9 | 5.7 ± 0 | 42.925 ± 1.8 | 17.51 ± 0.16 | 0.82 ± 0.01 | 2.985 ± 3.63 | 0.52 ± 0.47 | 21.23 ± 0.38 |
| AMP10 | 6.54 ± 0.57 | 43.74 ± 1.8 | 4.83 ± 0.01 | 0.24 ± 0.07 | 0.5 ± 0.61 | 1.61 ± 1.4 | 21.03 ± 6.14 |
| AMP11 | 6.20 ± 0.16 | 57.71 ± 17.72 | 16.17 ± 12.31 | 0.64 ± 0.32 | 2.23 ± 1.51 | 1.54 ± 2.15 | 23.20 ± 6.33 |
| AMP12 | 5.93 ± 0.34 | 57.98 ± 0.53 | 13.74 ± 4.84 | 0.76 ± 0.19 | 2.03 ± 0.26 | 2.19 ± 3.1 | 17.72 ± 1.9 |
| AMP13 | 5.97 ± 1.21 | 70.86 ± 10.66 | 22.83 ± 9.59 | 0.93 ± 0.06 | 3.74 ± 1.62 | 3.13 ± 2 | 24.12 ± 8.61 |
| TZP14 | 4.86 ± 0.58 | 62.55± 7.57 | 28.96 ± 5.3 | 0.79 ± 0.09 | 1.83 ± 1.6 | 2.00 ± 0.91 | 36.91 ± 8.01 |
| TZP15 | 4.92 ± 0.71 | 64.33 ± 2.79 | 21.96 ± 1.11 | 0.7 ± 0.05 | 4.03 ± 4.46 | 2.29 ± 1.87 | 31.51 ± 3.25 |

## *Values show the averages and standard deviations from the individual samples used for each pool.

## **Table S3**: Soil enzymatic activities* from circumpolar arctic sampling sites.

| **Sample** | **Exoglucanase (nmol g-1 dm h-1)** | **Endochitinase (nmol g-1 dm h-1)** | **Exochitinase (nmol g-1 dm h-1)** | **Protease (nmol g-1 dm h-1)** | **Phenoloxidase (nmol g-1 dm h-1)** | **Peroxidase (nmol g-1 dm h-1)** | **actual_Protease (nmol g-1 dm h-1)** | **X_actual_Cellulase (nmol g-1 dm h-1)** |
| --- | --- | --- | --- | --- | --- | --- | --- | --- |
| CHP1 | 208.04 ± 110.33 | 84.34 ± 23.82 | 1001.08 ± 207.26 | 360.01 ± 116.7 | 2786.49 ± 934.2 | 8280.59 ± 3231 | 3.09 ± 1.71 | 81.68 ± 33.08 |
| CHP2 | 224.3 ± 97.96 | 48.375 ± 29.98 | 857.48 ± 549.98 | 728.09 ± 245.62 | 3135.89 ± 800.48 | 11163.95 ± 2579.13 | 3.35 ± 2.68 | 78.10 ± 64.67 |
| CHP3 | 344.81 ± 66.88 | 116.42 ± 62.27 | 1247.60 ± 421 | 670.49 ± 342.52 | 2143.50 ± 1725.07 | 6242.05 ± 4670.94 | 2.88 ± 1.11 | 95.55 ± 96.78 |
| CHP4 | 33.54 ± NA | 14.96 ± NA | 257.53 ± NA | 117.04 ± NA | 826.33 ± NA | 3135.75 ± NA | 0.45 ± NA | 10.45 ± NA |
| CHP5 | 75.8 ± NA | 19.43 ± NA | 97.15 ± NA | 123.95 ± NA | 2206.8 ± NA | 7542.88 ± NA | 0.12 ± NA | 11.78 ± NA |
| ZKP6 | 472.33 ± 153.12 | 44.24 ± 34.61 | 692.55 ± 212.03 | 691.42 ± 75.54 | 2245 ± 520.26 | 7622.08± 1606.3 | 1.20 ± 0.67 | 42.83 ± 11.53 |
| ZKP7 | 266.7 ± NA | 17.39 ± NA | 378.48 ± NA | 603.38 ± NA | 1888.15 ± NA | 6102.38 ± NA | 0.68 ± NA | 27.54 ± NA |
| ZKP8 | 625.52 ± 237.36 | 270.94 ± 49.96 | 1273.47 ± 800.62 | 1710.19 ± 892.24 | 7290.48 ± 2236.88 | 20467 ± 5657.79 | 2.55 ± 2.2 | 390.81 ± 505.08 |
| ZKP9 | 325.80 ± 221.16 | 114.69 ± 66.4 | 443.23 ± 5.54 | 452.77 ± 7.81 | 688.05 ± 67.27 | 1959.86 ± 448.76 | 3.44 ± 2.06 | 48.64 ± 1.39 |
| AMP10 | 66.59 ± 68.13 | 26.05 ± 12.55 | 355.44 ± 144.7 | 89.50± 27.73 | 834.95 ± 1004.31 | 1533.65 ± 2295.74 | 1.54 ± 1.17 | 12.39 ± 3.27 |
| AMP11 | 123.27 ± 66.91 | 78.81 ± 28.08 | 608.66 ± 301.64 | 259.12 ± 186.27 | 507.62 ± 209.55 | 1324.27± 598.85 | 3.96 ± 3.47 | 62.07 ± 63.56 |
| AMP12 | 474.03 ± 140.62 | 130.05 ± 54.2 | 902.07 ± 234.07 | 282.59 ± 53.27 | 678.26 ± 143.44 | 1065.53 ± 67.61 | 2.56 ± 0.73 | 101.97 ± 45.76 |
| AMP13 | 233.24 ± 23.24 | 69.65 ± 43.97 | 979.07 ± 624.14 | 390.02 ± 185.97 | 1197.03 ± 100.03 | 2679.09 ± 257.83 | 6.06 ± 6.2 | 274.91 ± 192.6 |
| TZP14 | 465.68 ± 527.11 | 60.91 ± 49.2 | 1213.89 ± 630.66 | 267.13 ± 38.41 | 1217.64 ± 16.48 | 2829.11 ± 551.11 | 3.25 ± 0.69 | 272.78 ± 185.43 |
| TZP15 | 396.78 ± 187.92 | 89.68 ± 53.84 | 1161.23 ± 574.22 | 187.68 ± 54.84 | 2072.07 ± 562.02 | 5671.77 ± 1112.3 | 3.40 ± 0.56 | 289.91 ± 83.29 |

## *Values show the averages and standard deviations from the individual samples used for each pool.

**
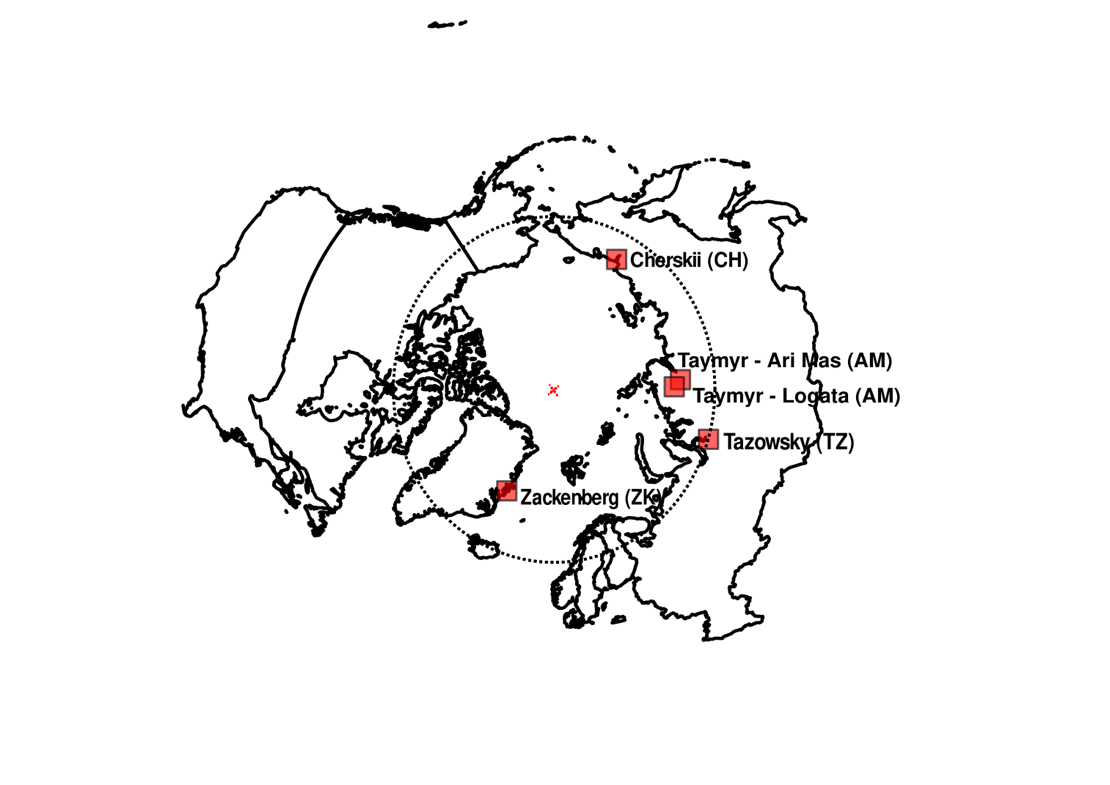
**

**Figure S1: Map of the sampled geographic locations across the circumpolar arctic.**


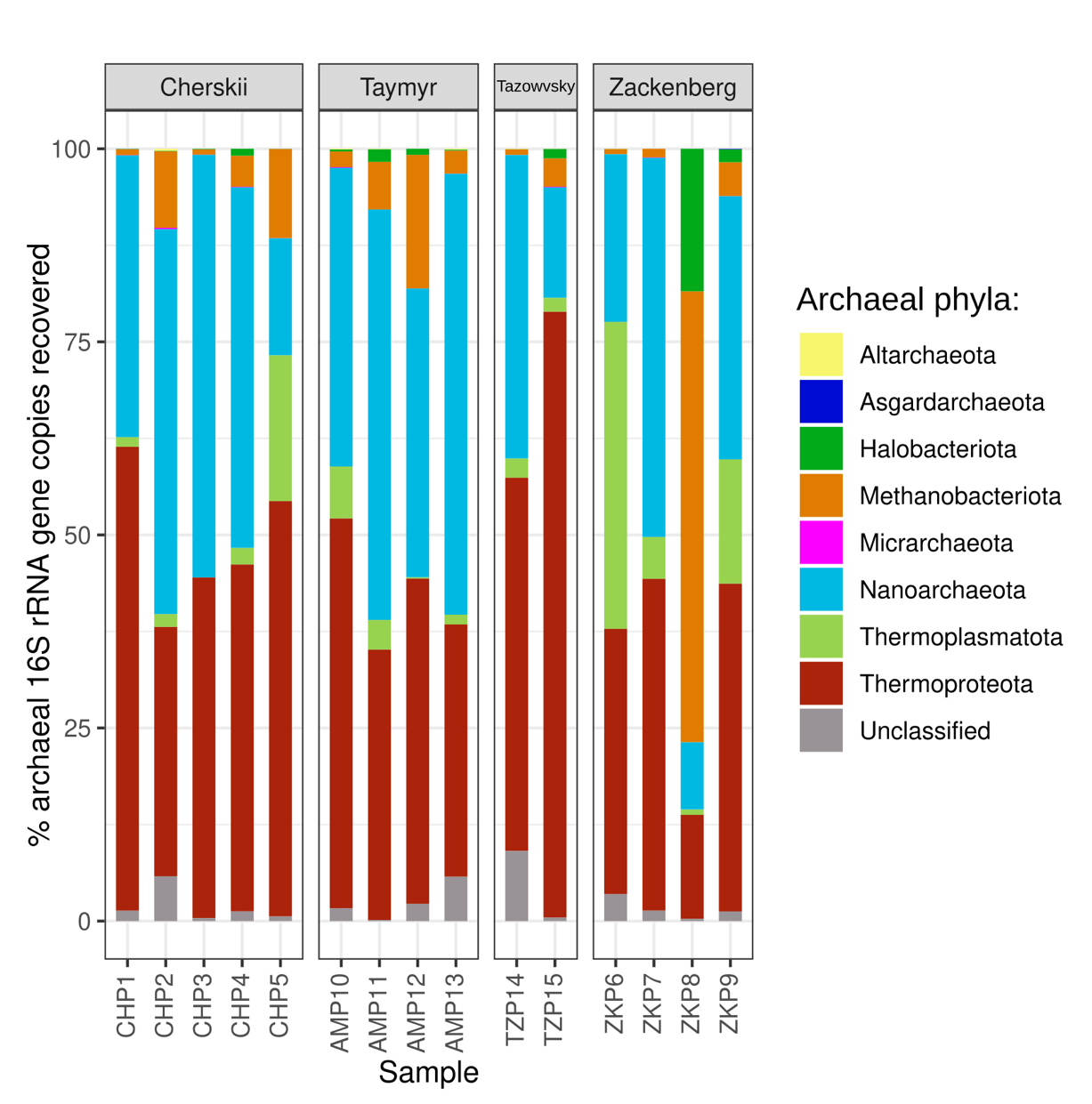


##

**Figure S2: Archaeal community at the phylum level in the different circumpolar arctic locations.** Barplots show the relative abundances of all archaeal AVSs. Reads were normalized to the total count of archaeal reads.


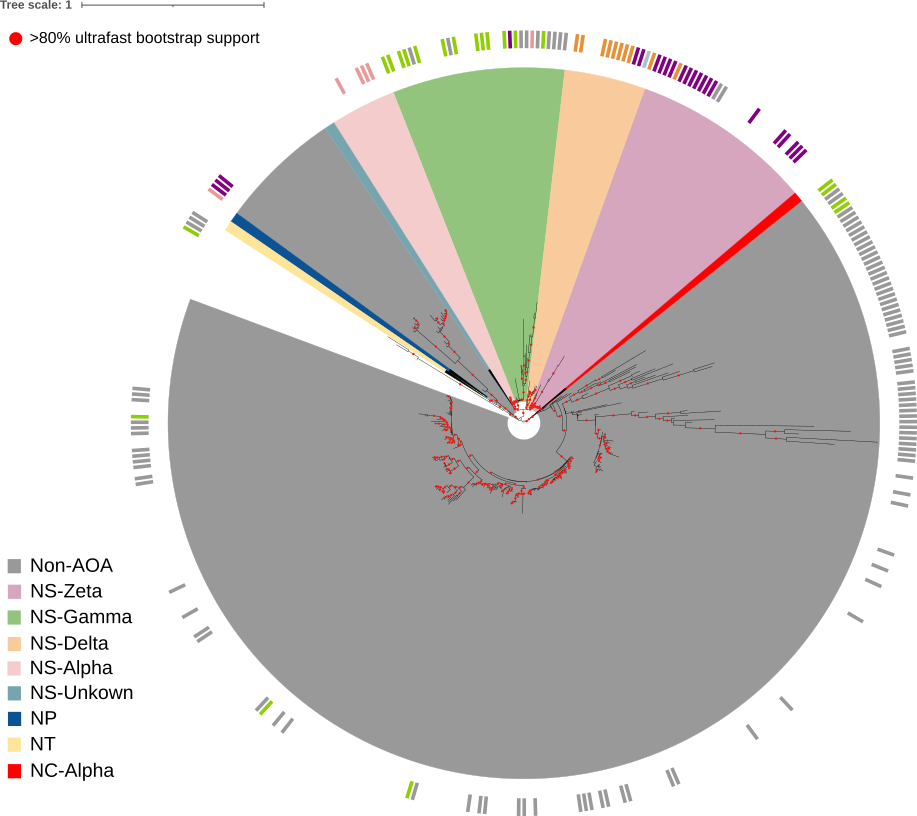


**Figure S3:** **16S rRNA gene phylogeny including Nitrosphaerales ASVs from this study.** Phylogenetic reconstruction based on full 16S rRNA gene sequences from *Nitrososphaerales*, including ASVs from this study. The colored boxes on the outer circle represent the classification of the ASVs obtained in this study into *amoA* gene-based clades using the 16S-to-*amoA* correspondence database (Wang et al. 2021) as described in Materials and methods.


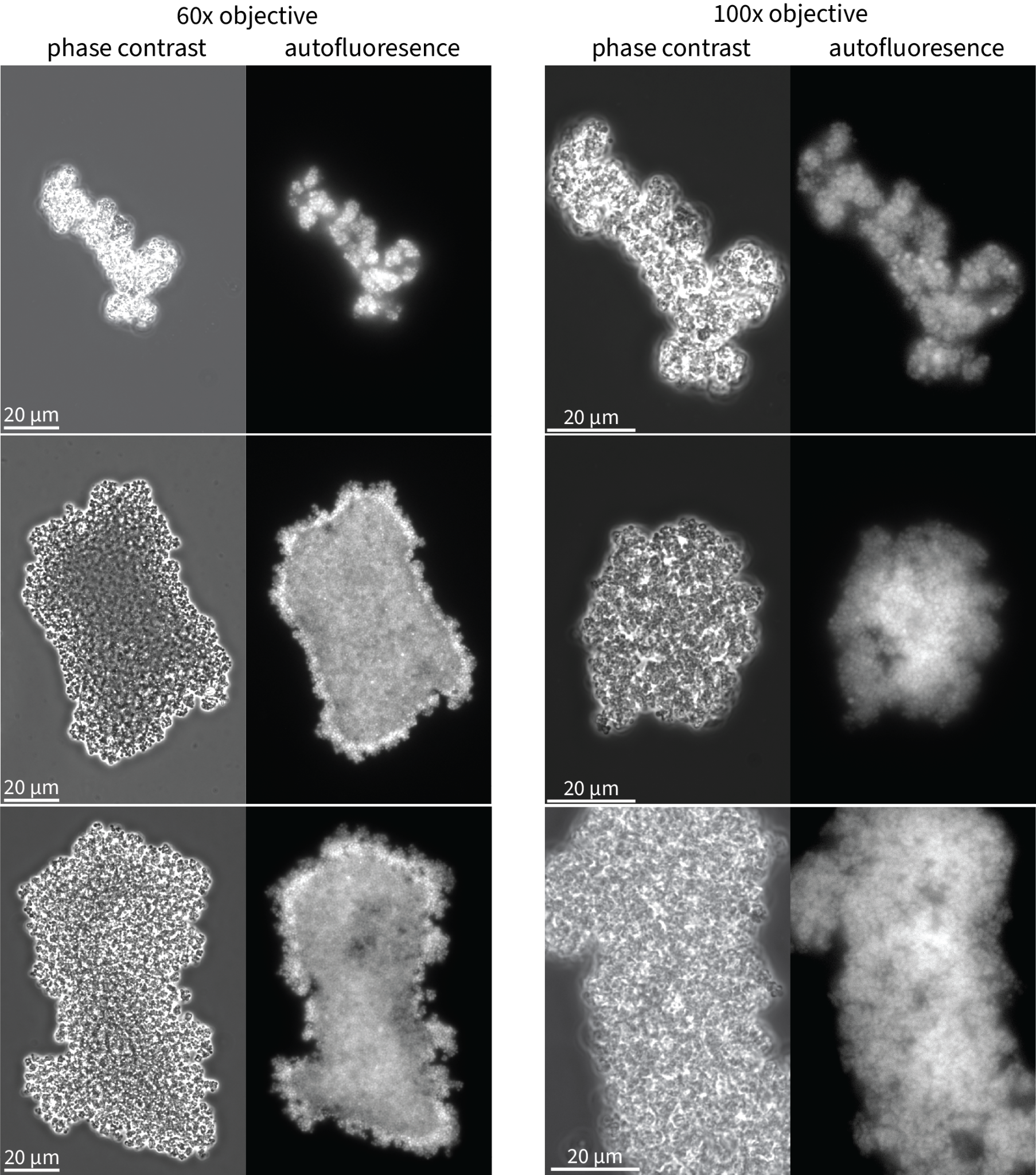
**Figure S4: Phase-contrast and fluorescence microscopy of aggregates of *Ca*. Nitrosocosmicus arcticus.** Autofluorescence of *Ca*. N. arcticus due to expression of cofactor F420 arcticus was imaged using a standard DAPI filter cube.


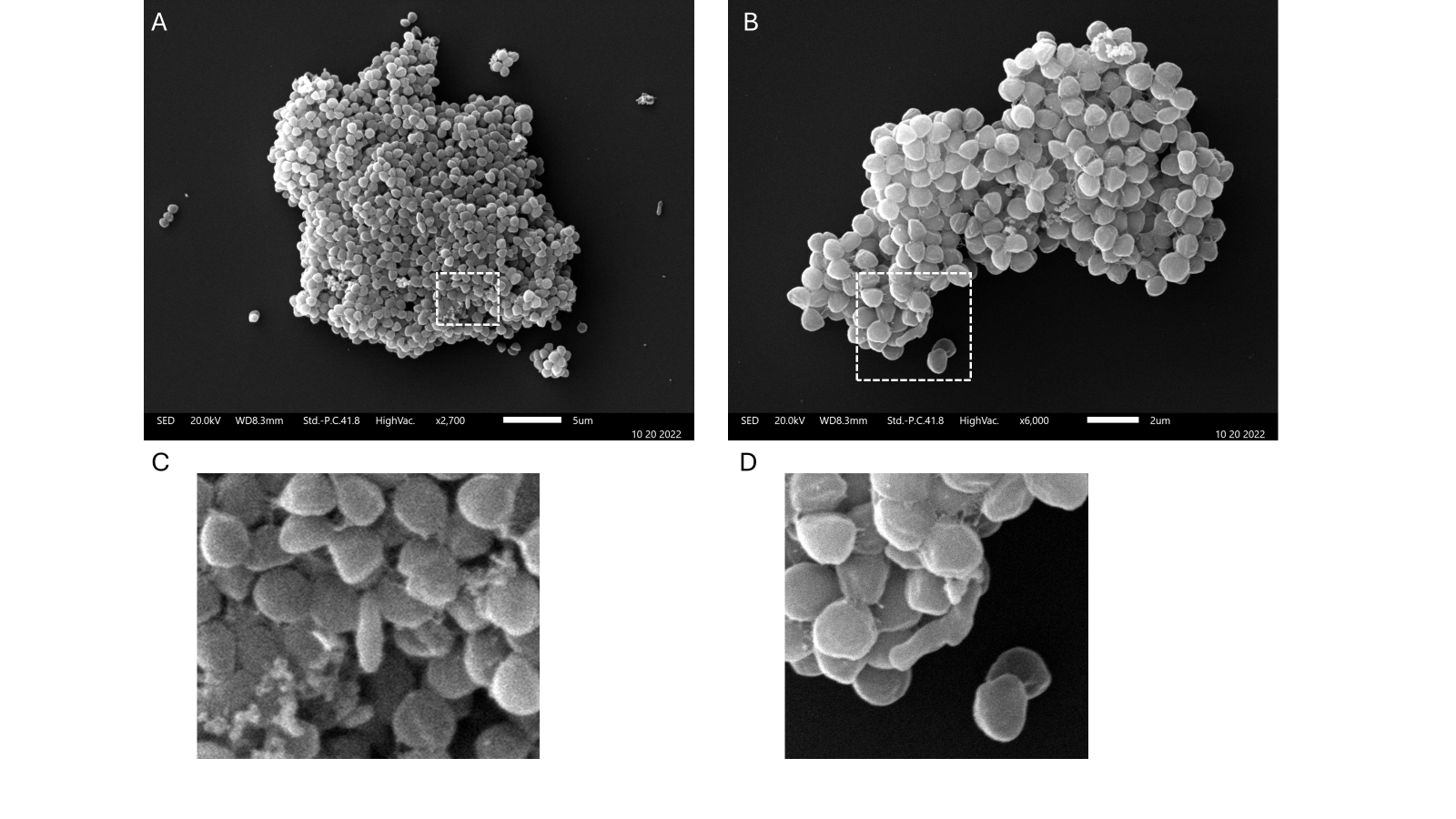


**Figure S5: Scanning electron microscopy (SEM) of bacterial contaminants bound to aggregates of *Ca*. Nitrosocosmicus arcticus**


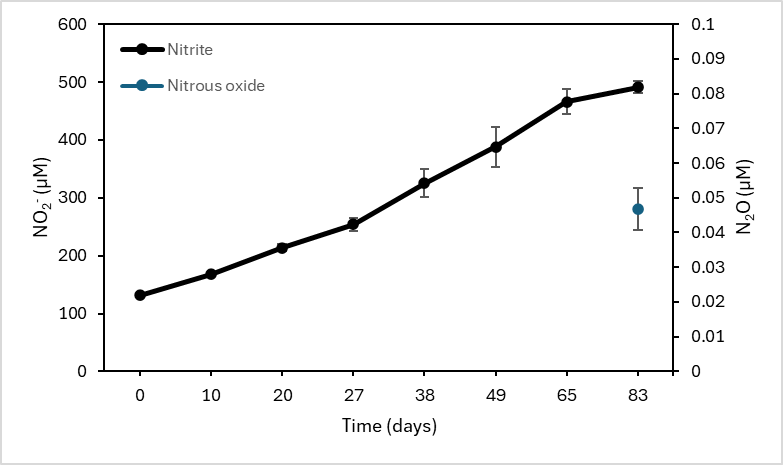


## **Figure S6: N_2_O yields of *Ca.* N. arcticus.**

Greenhouse gas emissions are hypothesized to increase with rising temperatures of arctic soils due to climate change. The pure culture of *Ca.* N. arcticus produced ~0.05 µM of N_2_O at a nitrite concentration of ~500 µM.

# 
